# The Role of Hyperthermia in Potentiation of Anti-Angiogenic Effect of Cisplatin and Resveratrol in Mice Bearing Solid Form of Ehrlich Ascites Tumour

**DOI:** 10.3390/ijms241311073

**Published:** 2023-07-04

**Authors:** Darko Kučan, Nada Oršolić, Dyana Odeh, Snježana Ramić, Boris Jakopović, Jelena Knežević, Maja Jazvinšćak Jembrek

**Affiliations:** 1 Division of Abdominal Surgery and Organ Transplantation, Department of Surgery, University Hospital Merkur, Zajčeva 19, 10000 Zagreb, Croatia; dkucan@gmail.com; 2 Division of Animal Physiology, Faculty of Science, University of Zagreb, Rooseveltov trg 6, 10000 Zagreb, Croatia; dyana.odeh@biol.pmf.hr; 3 Department of Pathology, University Cancer Hospital, Sestre Milosrdnice University Hospital Centre, Ilica 197, 10000 Zagreb, Croatia; snjezana.ramic@gmail.com; 4 Dr Myko San—Health from Mushrooms Co., Miramarska Cesta 109, 10000 Zagreb, Croatia; borisjakopovic@yahoo.com; 5 Division of Molecular Medicine, Ruđer Bošković Institute, Bijenička Cesta 54, 10000 Zagreb, Croatia; jknezev@irb.hr (J.K.); maja.jazvinscak.jembrek@irb.hr (M.J.J.); 6 School of Medicine, Catholic University of Croatia, Ilica 242, 10000 Zagreb, Croatia

**Keywords:** resveratrol, Ehrlich solid tumour, mice, angiogenesis, cisplatin, whole body hyperthermia treatment, HSP70/HSP90

## Abstract

The aim of this study was to investigate the therapeutic potential of resveratrol in combination with cisplatin on the inhibition of tumour angiogenesis, growth, and macrophage polarization in mice bearing the solid form of an Ehrlich ascites tumour (EAT) that were exposed to whole-body hyperthermia treatment. In addition, we investigated whether a multimodal approach with hyperthermia and resveratrol could abolish cisplatin resistance in tumour cells through the modulation of histone deacetylase (HDAC) activity and levels of heat shock proteins (HSP70/HSP90) and contribute to the direct toxicity of cisplatin on tumour cells. The tumour was induced by injecting 1 × 10^6^ EAT cells subcutaneously (*sc*) into the thighs of Balb/c mice. The mice were treated with resveratrol *per os* for five consecutive days beginning on day 2 after tumour injection and/or by injecting cisplatin intraperitoneally (*ip*) at a dose of 2.5 mg/kg on days 10 and 12 and at a dose of 5 mg/kg on day 15. Immediately thereafter, the mice were exposed to systemic hyperthermia for 15 min at a temperature of 41 °C. The obtained results showed that the administration of resveratrol did not significantly contribute to the antitumour effect of cisplatin and hyperthermia, but it partially contributed to the immunomodulatory effect and to the reduction of cisplatin toxicity and to a slight increase in animal survival. This treatment schedule did not affect microvessel density, but it inhibited tumour growth and modulated macrophage polarization to the M1 phenotype. Furthermore, it abolished the resistance of tumour cells to cisplatin by modulating HDAC activity and the concentration of HSP70 and HSP90 chaperones, contributing to the increased lifespan of mice. However, the precise mechanism of the interaction between resveratrol, cisplatin, and hyperthermia needs to be investigated further.

## 1. Introduction

Angiogenesis is the process of the formation of new blood vessels and is one of the main factors that promote tumour growth. Angiogenesis occurs as a response to hypoxia, which, together with a lack of essential nutrients, increases the expression of inflammatory signals and cytokines that recruit blood vessel cells to create a network of new tumour blood vessels [1]. Hypoxia inducible factor-1α (HIF-1α) plays a key role in tumour adaptation to the hypoxic microenvironment by stimulating the production of pro-angiogenic factors, such as vascular endothelial growth factor (VEGF) and matrix melalloproteinases MMP-2 and MMP-9, and by inducing the expression of enzymes participating in anaerobic metabolism. The process of angiogenesis includes several steps: the release of angiogenic factors, the release of proteolytic enzymes, the migration of endothelial cells towards the tumour, and the proliferation and maturation of endothelial cells into a new blood vessel. Among numerous factors involved in the process of angiogenesis, VEGF is considered the critical factor of tumour neovascularization and tumour progression in vivo because it stimulates the proliferation of endothelial cells and remodels the extracellular matrix of blood vessels by upregulating proteinases such as matrix metalloproteinases (MMPs).

Tumours with higher angiogenic potential show higher metastatic capacity and aggressiveness [2]. MMPs play an important role in the process of tumour angiogenesis by participating in the degradation of the extracellular matrix and the basement membrane of the postcapillary venules, facilitating cell invasion. The proteolytic activity of this family of enzymes is also responsible for capillary elongation, the formation of the vascular lumen, and the remodelling of the extracellular matrix. Hence, MMPs serve as biomarkers and potential therapeutic targets in the treatment of cancer and other vascular diseases [3,4]. Regarding the role of angiogenesis in antitumour therapy, there is a great interest in the development of anti-angiogenic therapeutic approaches capable of inhibiting tumour vascularization and preventing its further growth and spreading. Common approaches include the use of anti-angiogenic drugs that target and block the activities of pro-angiogenic factors. Given that their effectiveness is still debated, new approaches are directed into the combined use of anti-angiogenic agents with chemotherapy or immunotherapy [5]. In the inhibition of tumour proliferation and growth, the cytostatic cisplatin is often used as one of the most effective chemotherapeutics in the elimination of tumour cells, but also as a potential immunomodulator. A combination of cisplatin and heat treatment has synergistic effects and can induce cancer cell death by stimulating several physiological responses, including the anti-angiogenic effect. The anti-angiogenic effect is achieved through the suppression of VEGF expression originating from the tumour, thereby inhibiting the proliferation of endothelial cells and the remodelling of the extracellular matrix into blood vessels and stimulating the immune response through the activation of immune cells [6,7,8]. It has been shown that chronic and acute exposure to cisplatin promotes the intratumoural accumulation of T cells, increases the immunogenicity of cancer cells, and regulates immunogenic cell markers (calreticulin), MHC class I molecules, and molecules involved in antigen processing and presentation [8]. Cisplatin also increases the tumour mutational load and the potential for neoepitope formation. Platinum-based chemotherapy is used in the treatment of various types of tumours, and it is especially recommended as the first line of treatment for the majority of advanced and inoperable cancers [9]. Cytotoxic activity of cisplatin is based on the induction of oxidative stress, DNA damage, and the activation of signalling pathways that ultimately results in apoptosis. Besides killing tumour cells, cisplatin acts on normal, healthy cells, leading to neurotoxicity, the suppression of bone marrow activity, hepatotoxicity, nephrotoxicity, and tumour cell resistance to the drug [6,7]. Chemicals, high temperature, free radicals, and mechanical stress can induce a heat shock protein (HSP) response whose functional activity can be regulated by HDAC inhibitors. HDAC inhibitors produce a marked inhibition of HIF-1α expression and are currently in clinical trials, partly due to their potent anti-angiogenic effects [10].

With the aim of improving treatment options for cancer, there is a growing interest in the scientific community about the potential therapeutics originating from biogenic sources with HDAC inhibitory activities. Based on that, we hypothesised that combinations of natural compounds and chemotherapeutics could enhance the sensitivity of cancer cells, overcome resistance, increase the cytotoxicity, improve the treatment outcome, and reduce the cisplatin-induced side effects. An example of such a compound is resveratrol, a phytoestrogen and polyphenol with antioxidant, anti-inflammatory, cardioprotective, and antitumour properties. The administration of resveratrol can reverse multidrug resistance and in combination with cisplatin has an additive and/or synergistic effect in increasing the chemosensitivity of cancer cells, reducing cytotoxic effects on healthy cells and modulating the immune response in the tumour microenvironment by regulating cytokines/chemokines secretion, immune-checkpoints/MHCI (major histocompatibility complex 1), the nuclear factor-κB (NF-κB) signalling pathway, the AKT-mammalian target of rapamycin (mTOR) signalling, and the activation of receptors on natural killer (NK) cells [11,12]. Yang et al. [12] showed that resveratrol in combination with cisplatin synergistically inhibits the viability of MDA231 breast cancer cells, as well as their migration and invasion in the MDA231 xenograft model, probably by modifying epithelial to the mesenchymal transition (EMT) program and through the regulation of PI3K/AKT, Smad, NF-B, JNK, and ERK expression in tumour tissue. Resveratrol was also found to enhance the inhibitory effects of cisplatin on cancer cell proliferation, the induction of apoptosis, cytochrome c release, and abnormal expression of Bcl-2 and Bax proteins [9]. In addition, Zhang et al. [13] showed that pre-treatment with resveratrol reduces the hypoxia-induced expression of HIF-1α and VEGF in human tongue squamous cell carcinoma and hepatoma cells and suggested that HIF-1α/VEGF could be promising drug targets in the development of an effective chemopreventive and anticancer therapy. Furthermore, the reduced level of HIF reduces the expression of the GLUT-1 transporter and consequently decreases glucose uptake into tumour cells, lactate production, Akt and mTOR signalling, and cell viability, depending on the dose and time. It was further shown that resveratrol can inhibit all eleven human HDACs of classes I, II, and IV in a dose-dependent manner and that HDAC inhibitors could have multiple mechanisms of inducing cell growth arrest, angiogenesis, and cell death [14]. In addition, resveratrol promotes acetylation of p53 and thus increases the expression of p53 target genes that participate in cell death and growth arrest [11,12,13]. According to Narita et al. [15], hyperthermia and HDAC inhibitors can be a possible modality for cancer therapy because specific inhibitors of HDAC could enhance the antitumour effects of hyperthermia without any severe side effects. 

Hence, the aim of this study was to investigate the effects of the combined treatment with cisplatin and resveratrol on metalloproteinase activity, angiogenesis, tumour growth, and survival in mice bearing the solid form of Ehrlich’s ascites tumour. This model is a suitable model in tumour biology and is useful for studying tumour pathogenesis and the development of antitumour drugs [6]. Furthermore, we were interested if resveratrol, applied with or without the whole-body hyperthermia treatment, can enhance the effect of cisplatin by modulating HDAC activity and levels of HSP70 and HSP90 and thus contribute to the direct toxicity on tumour cells, the inhibition of angiogenesis and tumour growth, and consequently the increased lifespan of mice.

## 2. Results

### 2.1. Combination of Resveratrol, Cisplatin, and Hyperthermia Improves Survival Rate and Induces Regression of Tumour Volume in Balb/c Mice Bearing the Solid Form of EAT

**Figure 1:**
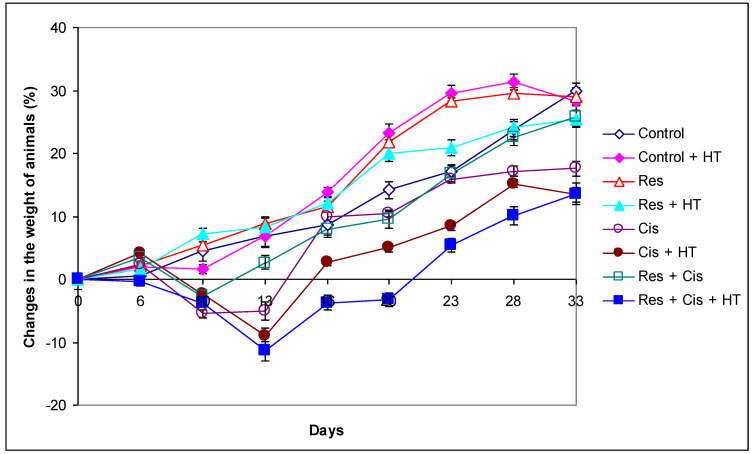
Weight changes of mice bearing the solid form of EAT treated with resveratrol, cisplatin, and their combination in physiological and hyperthermic conditions. After subcutaneous (*sc*) injection of 1 × 10^6^ EAT cells, mice (N = 10 per group) were treated with a dose of 50 mg/kg resveratrol during five consecutive days starting from the second day after injection, while cisplatin was injected intraperitoneally (*ip*) at a dose of 2.5 mg/kg on days 10 and 12 and at a dose of 5 mg/kg on day 15. Groups treated with hyperthermia were exposed to systemic hyperthermia lasting for 15 min at a temperature of 41 °C. Obtained results are presented as Mean ± SE. Statistical difference between groups per day: 13. Control vs. Cis + HT (*p* ≤ 0.05); Control vs. Res + Cis + HT (*p* ≤ 0.05); Res vs. Cis (*p* ≤ 0.05); Res vs. Cis + HT (*p* ≤ 0.05); Res vs. Res + Cis (*p* ≤ 0.01); Res vs. Res + Cis + HT (*p* ≤ 0.01); Res + HT vs. Cis (*p* ≤ 0.01); Res + HT vs. Cis + HT (*p* ≤ 0.05); Res + HT vs. Res + Cis (*p* ≤ 0.01); Res + HT vs. Res + Cis + HT (*p* ≤ 0.001); 16. Control vs. Res + Cis (*p* ≤ 0.05); Control vs. Res + Cis + HT (*p* ≤ 0.05); Res vs. Cis + HT *p* ≤ 0.05); Res vs. Res + Cis (*p* ≤ 0.05); Res vs. Res + Cis + HT (*p* ≤ 0.05); Res + HT vs. Cis + HT (*p* ≤ 0.05); Res + HT vs. Res + Cis (*p* ≤ 0.05); Res + HT vs. Res + Cis + HT (*p* ≤ 0.05); 23. Res + HT vs. Cis (*p* ≤ 0.05); Res + HT vs. Cis + HT (*p* ≤ 0.01); Res + HT vs. Res + Cis + HT (*p* ≤ 0.001). Abbreviations: Res—resveratrol solution at a dose of 50 mg/kg; Cis—cisplatin at doses of 2.5 and 5 mg/kg; Res + Cis—resveratrol solution at a dose of 50 mg/kg and cisplatin at doses of 2.5 and 5 mg/kg; HT—hyperthermia; SE—Standard error.

**Figure 2:**
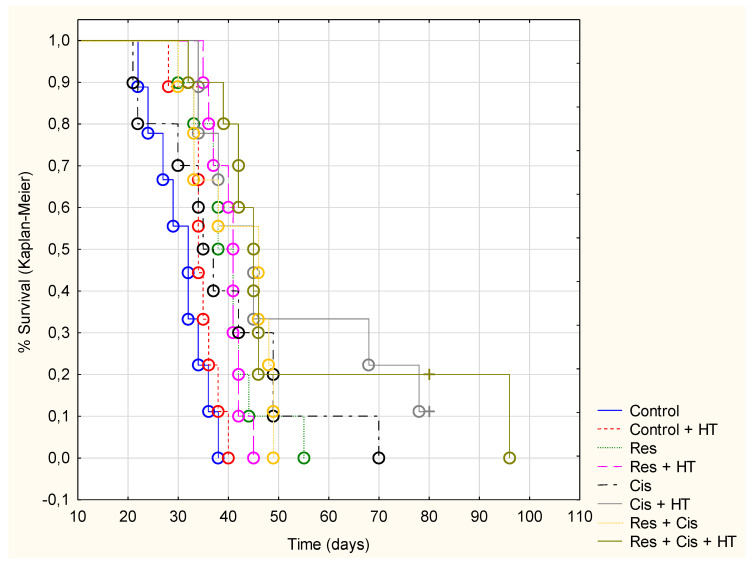
Kaplan–Meier survival assay of mice bearing the solid form of EAT treated with resveratrol, cisplatin, and their combination in physiological and hyperthermic conditions. After subcutaneous (*sc*) injection of 1 × 10^6^ EAT cells, mice (N = 10 per group) were treated with a dose of 50 mg/kg resveratrol during five consecutive days starting from the second day after injection, while cisplatin was injected intraperitoneally (*ip*) at a dose of 2.5 mg/kg on days 10 and 12 and at a dose of 5 mg/kg on day 15. Groups treated with hyperthermia were exposed to systemic hyperthermia lasting for 15 min at a temperature of 41 °C. Abbreviations: Res—resveratrol solution at a dose of 50 mg/kg; Cis—cisplatin at doses of 2.5 and 5 mg/kg; Res + Cis—resveratrol solution at a dose of 50 mg/kg and cisplatin at doses of 2.5 and 5 mg/kg; HT—hyperthermia.

**Figure 3:**
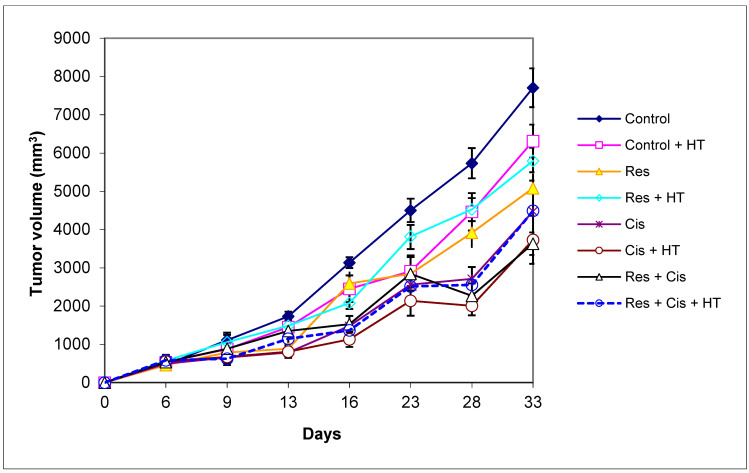
Tumour volume changes of mice bearing the solid form of EAT treated with resveratrol, cisplatin, and their combination in physiological and hyperthermic conditions. After subcutaneous (*sc*) injection of 1 × 10^6^ EAT cells, mice (N = 10 per group) were treated with a dose of 50 mg/kg resveratrol during five consecutive days starting from the second day after injection, while cisplatin was injected intraperitoneally (*ip*) at a dose of 2.5 mg/kg on days 10 and 12 and at a dose of 5 mg/kg on day 15. Groups treated with hyperthermia were exposed to systemic hyperthermia lasting for 15 min at a temperature of 41 °C. Obtained results are presented as Mean ± SE. Statistical difference between groups per day: 13. Control vs. Res + Cis + HT (*p* ≤ 0.05); 28. Control vs. Cis + HT (*p* ≤ 0.05); Control + HT vs. Cis + HT (*p* ≤ 0.05); Cis + HT vs. Res + HT (*p* ≤ 0.05). Abbreviations: Res—resveratrol solution at a dose of 50 mg/kg; Cis—cisplatin at doses of 2.5 and 5 mg/kg; Res + Cis—resveratrol solution at a dose of 50 mg/kg and cisplatin at doses of 2.5 and 5 mg/kg; HT—hyperthermia; SE—Standard error.

In patients and animals, cisplatin causes prominent side effects that increase morbidity and impair quality of life. Currently, around 40 side effects of cisplatin have been reported [16], including gastrointestinal tract disorders associated with the acute and delayed nausea and vomiting, gastric stasis, reduced food intake, and subsequent weight loss. In order to study the gastrointestinal side effects of cisplatin, the body weight of each mouse was measured from day 0 to day 33 during the experimental period. Although the cisplatin was applied at relatively low doses (2.5 or 5 mg/kg), the marked decrease of animal weight was observed in groups exposed to cisplatin and hyperthermia with and/or without resveratrol (Res+ Cis + HT and Cis + HT), which indicates the more pronounced toxic effect of cisplatin in hyperthermic conditions (Figure 1). In the Control, Control + HT, Res, Res + HT, or Cis + Res groups there was no change of animal weight, indicating that resveratrol reduces the toxic effects of cisplatin under physiological conditions. Weight loss was not only associated with cisplatin toxicity, but also with tumour growth inhibition and increased animal survival (Figure 2 and Figure 3).

Treatment with cisplatin and resveratrol under hyperthermic conditions increased the lifespan by about 68% and reduced tumour growth by about 50% compared to the Control group (Table 1, Figure 2). Increased life span (ILS%) for the Cis treated group was 27.79%, while Cis + HT increased life span by 67.90% in relation to the Control group. The group treated with cisplatin on day 16 showed a 53% inhibition of tumour growth (TVI) compared to the Control, while the group treated with cisplatin and resveratrol on day 16 showed an inhibition of 51% compared to the Control group. On day 33, the group treated with cisplatin showed a 41% TVI compared to the Control, while the group treated with cisplatin and resveratrol showed an inhibition of 52% compared to the Control group. The Cis + HT group showed the highest percentage of inhibition compared to other groups (approximately 64% and 52% on day 16 and 33, respectively). For the group treated with Res + Cis + HT, TVI was 56.50% compared to the Control on day 16 and 42% on day 33. According to the obtained data, Cis in combination with HT demonstrated a better inhibitory effect on tumour growth (Figure 3), but also greater toxicity, which together reduced the survival of the animals (Figure 2), while in the combination Res + Cis + HT, resveratrol could partially have a protective or immunomodulatory effect. However, it should be emphasized that there was no difference between these two groups (Cis + HT and Res + Cis + HT) in mean survival time and %ILS. The obtained values for mean survival time were 51.11 ± 6.28 and 51.30 ± 6.37 and for %ILS 67.90% and 68.52%, respectively (Table 1).

Preventive *ig* treatment of animals with resveratrol (50 mg/kg) or therapeutic treatment with cisplatin (2.5 or 5 mg/kg) under normal and hyperthermic conditions significantly reduced the tumour volume of mice bearing the solid Ehrlich tumour compared to controls (Control and Control + HT). Table 1 shows the inhibition of the tumour volume on day 16 (immediately after therapy) and day 33 (up to which the tumour volume was monitored) compared to physiological and hyperthermic control groups.

Preventive treatment with resveratrol has reinforced the delay of tumour growth in mice treated with cisplatin and increased the survival of animals (Figure 2 and Figure 3).

The survival rate of mice after treatment with test components in physiological and hyperthermic conditions was shown by the Kaplan–Meier method (Figure 2) and analysed by the long-rank test (Table 2). According to the survival rate, the best overall survival of mice was observed following treatment with Res + Cis + HT in relation to the Control or Control + HT groups (*p* = 0.00233 and *p* = 0.00506, respectively).

The lack of difference between Cis + HT and Res + Cis + HT was also confirmed by the Kaplan–Meier analysis of survival; there was no statistical difference between these groups (*p* = 0.68082). In addition to the comparison with the control groups (Control and Control + HT, Table 2), we analysed the relationship between the other groups. The statistically significant difference was observed only between the groups Res vs. Res + Cis + HT (*p* = 0.04231) and Res + HT vs. Res + Cis + HT (*p* = 0.02158) (see Supplementary Material, Table S1).

### 2.2. Combination of Resveratrol, Cisplatin, and Hyperthermia Inhibits HDACs Level, Supresses HIF-1α Level, and Modulates HSP70 and HSP90 Expression in Balb/c Mice Bearing the Solid Form of EAT

Resveratrol as a natural product could partly affect the inhibition of tumour growth through epigenetic mechanisms by acting on HDACs. HDACs are enzymes involved in numerous steps of tumourigenesis, including the initiation of tumour growth, angiogenesis, metastasis, and numerous other processes of tumour progression [10,11,12,17]. Considering the important role of HDACs in tumourigenesis, especially in the regulation of angiogenesis and HSPs and HIF-1α activity, we further investigated whether the resveratrol could modulate HDAC activity and enhance the chemotherapeutic effect of cisplatin in normal and hyperthermic conditions acting as an epigenetic modulator.

**Figure 4:**
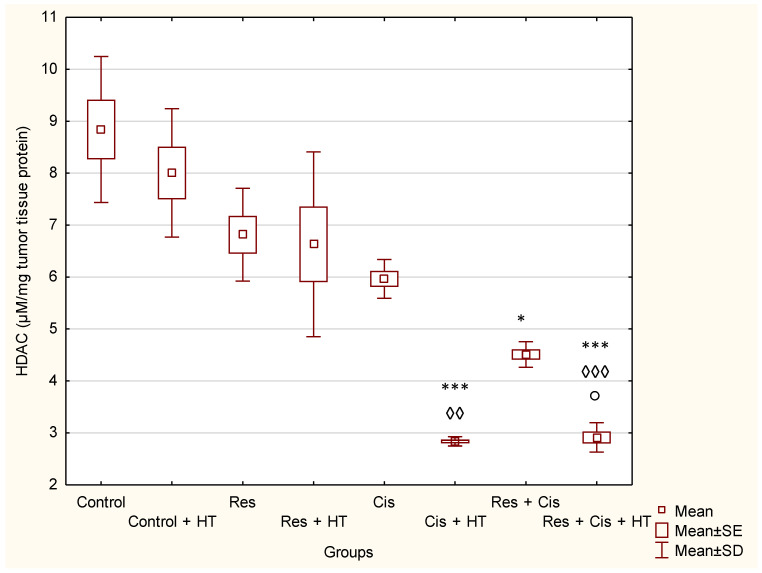
HDAC activity (µM/mg tumour tissue protein) in mice bearing the solid form of EAT treated with resveratrol, cisplatin, and their combination in physiological and hyperthermic conditions. After subcutaneous (*sc*) injection of 1 × 10^6^ EAT cells, mice (N = 5 per group) were treated with a dose of 50 mg/kg resveratrol during five consecutive days starting from the second day after injection, while cisplatin was injected intraperitoneally (*ip*) at a dose of 2.5 mg/kg on days 10 and 12 and at a dose of 5 mg/kg on day 15. Groups treated with hyperthermia were exposed to systemic hyperthermia lasting for 15 min at a temperature of 41 °C. Obtained results are presented as Mean ± SD (SE) of duplicate determination. * Significantly different in relation to Control (* *p* ≤ 0.05; *** *p* ≤ 0.001). ^◊^ Significantly different in relation to Control + HT (^◊◊^ *p* ≤ 0.01; ^◊◊◊^ *p* ≤ 0.001). ^○^ Significantly different in relation to Res (^○^ *p* ≤ 0.05). Abbreviations: Res—resveratrol solution at a dose of 50 mg/kg; Cis—cisplatin at doses of 2.5 and 5 mg/kg; Res + Cis—resveratrol solution at a dose of 50 mg/kg and cisplatin at doses of 2.5 and 5 mg/kg; HT—hyperthermia; SE—Standard error; SD—Standard deviation.

According to the obtained data, pre-treatment with resveratrol enhanced the cytotoxic effects of cisplatin on HDAC activity in comparison to the Control group. HDAC inhibition values were: 4.51 ± 0.10 (Res + Cis) vs. 8.84 ± 0.57 (Control), *p* < 0.05. However, hyperthermia further enhanced the inhibitory effect of cisplatin on HDAC activity. The HDAC inhibition values obtained were: 2.84 ± 0.04 (Cis + HT) and 2.91 ± 0.11 (Res + Cis + HT) vs. 8.84 ± 0.57 (Control), *p* < 0.001 for both groups (Figure 4).

Chaperones are usually classified according to their molecular weight. HSP70 and HSP90 chaperones participate in de novo folding and refolding of proteins. Thus, HSP90 participates in the posttranslational folding and stabilization of more than 200 client proteins involved in the regulation of those cell signals that promote tumour cell growth and radioresistance [18], while HSP70 protects cancer cells against apoptosis. In addition, the dual role of HSPs in antitumour immunity has been demonstrated [18]. 

**Figure 5:**
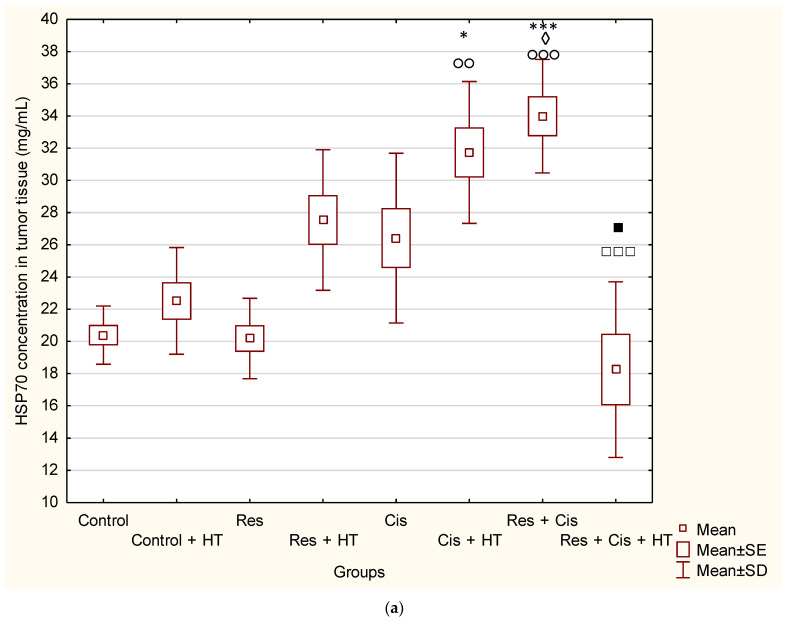

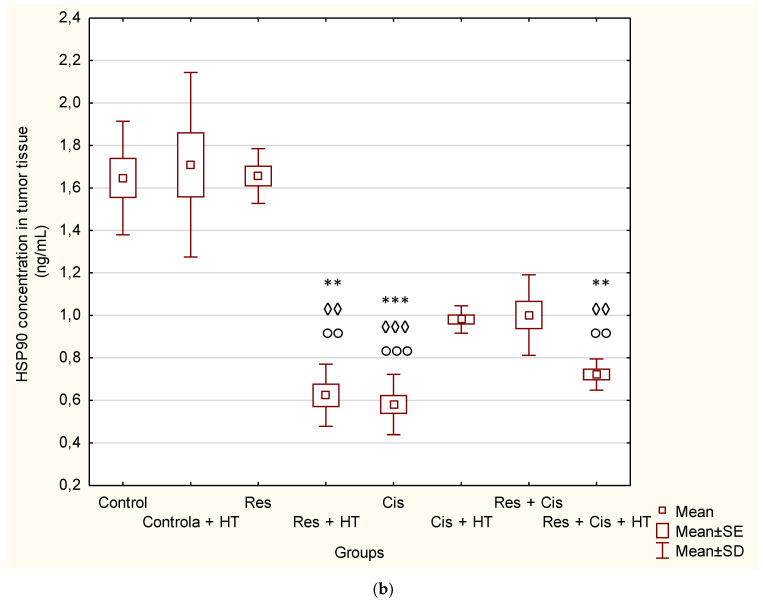
HSP70 (**a**) and HSP90 (**b**) concentrations in tumour tissue in mice bearing the solid form of EAT treated with resveratrol, cisplatin, and their combination in physiological and hyperthermic conditions. After subcutaneous (*sc*) injection of 1 × 10^6^ EAT cells, mice (N = 5 per group) were treated with a dose of 50 mg/kg resveratrol during five consecutive days starting from the second day after injection, while cisplatin was injected intraperitoneally (*ip*) at a dose of 2.5 mg/kg on days 10 and 12 and at a dose of 5 mg/kg on day 15. Groups treated with hyperthermia were exposed to systemic hyperthermia lasting for 15 min at a temperature of 41 °C. Obtained results are presented as Mean ± SD (SE) of duplicate determination. * Significantly different in relation to Control (* *p* ≤ 0.05; ** *p* ≤ 0.01; *** *p* ≤ 0.001). ^◊^ Significantly different in relation to Control + HT (^◊^ *p* ≤ 0.05; ^◊◊^ *p* ≤ 0.01; ^◊◊◊^ *p* ≤ 0.001). ^○^ Significantly different in relation to Res (^○○^ *p* ≤ 0.01; ^○○○^ *p* ≤ 0.001). ^□^ Significantly different in relation to Res + Cis (^□□□^ *p* ≤ 0.001). ^■^ Significantly different in relation to Cis + HT (^■^ *p* ≤ 0.05). Abbreviations: Res—resveratrol solution at a dose of 50 mg/kg; Cis—cisplatin at doses of 2.5 and 5 mg/kg; Res + Cis—resveratrol solution at a dose of 50 mg/kg and cisplatin at doses of 2.5 and 5 mg/kg; HT—hyperthermia; SE—Standard error; SD—Standard deviation.

Interestingly, levels of HSP70 (Figure 5a) were higher compared to the Control group, except for the Res and Res + Cis + HT groups. Concentrations of HSP70 were increased as follows: 66.69% (Res + Cis); 55.64% (Cis + HT); 35.06% (Res + HT); 29.55% (Cis); and 10.42% (Control + HT).

Treatment with Res + Cis showed the highest value of HSP70 compared to Control (*p* ≤ 0.001), Control + HT (*p* ≤ 0.05), and Res (*p* ≤ 0.001). The second highest value was observed for the treatment with Cis + HT (compared to Control *p* ≤ 0.05 and Res *p* ≤ 0.01). According to the obtained data, the group treated with Res + Cis + HT had only a slightly lower level of HSP-70 compared to the Control and Control + HT groups, which indicates greater sensitivity of tumour cells since the inhibition of HSP70 can enhance the chemotherapy-induced tumour-killing effect. This group showed a significant decrease of HSP70 concentration compared to Cis + HT *(p* ≤ 0.05) and Res (*p* ≤ 0.001).

HSP90 concentrations (Figure 5b) were reduced in all groups compared to the Control, except for the group treated with resveratrol; % inhibition from the highest value to the lowest were: 70.24% (Cis); 68.04% (Res + HT); 62.99% (Res + Cis + HT); 49.70% (Cis + HT); 48.64% (Res + Cis); 23.80% (Control + HT), and 0.22% (Res). Thus, the lowest value of HSP90 was observed in the Cis group compared to Control, Control + HT, and Res (*p* ≤ 0.001), followed by the values obtained in groups Res + HT and Res + Cis + HT (compared to Control, Control + HT, and Res *p* ≤ 0.01).

### 2.3. Combination of Resveratrol, Cisplatin, and Hyperthermia Suppresses Angiogenesis and Increases Tumouricidal Activity of Macrophages in Balb/c Mice Bearing the Solid Form of EAT

Hyperthermia causes numerous changes of cellular membranes, the cytoskeleton, the nucleus, DNA, RNA, proteins, and cell organelles, but also damages the microvasculature and suppresses the angiogenesis of malignant tumours. Angiogenesis is essential for tumour development and metastasis, and tumour-derived angiogenic factor(s) such as VEGF play(s) an important role in the formation of new vessels and tumour progression in vitro and in vivo.

**Figure 6:**
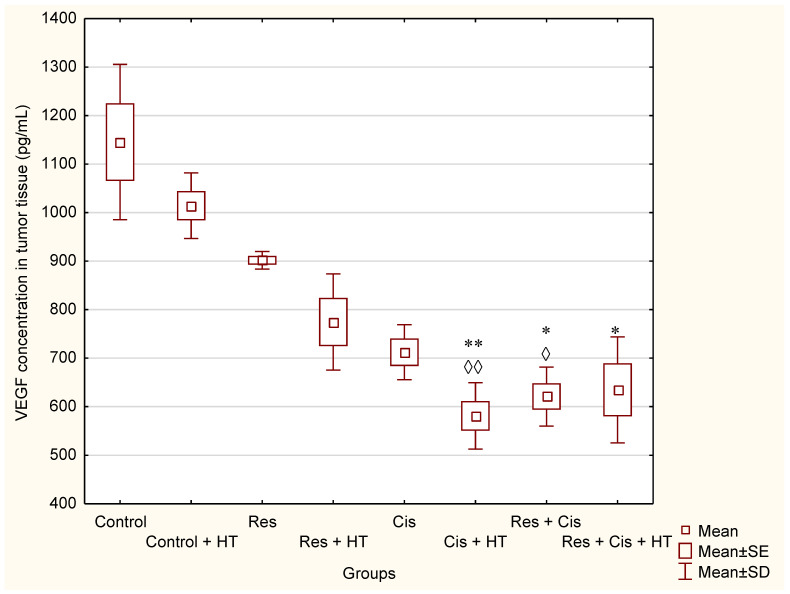
VEGF concentration in tumour tissue (pg/mL) in mice bearing the solid form of EAT treated with resveratrol, cisplatin, and their combination in physiological and hyperthermic conditions. After subcutaneous (*sc*) injection of 1 × 10^6^ EAT cells, mice (N = 5 per group) were treated with a dose of 50 mg/kg resveratrol during five consecutive days starting from the second day after injection, while cisplatin was injected intraperitoneally (*ip*) at a dose of 2.5 mg/kg on days 10 and 12 and at a dose of 5 mg/kg on day 15. Groups treated with hyperthermia were exposed to systemic hyperthermia lasting for 15 min at a temperature of 41 °C. Obtained results are presented as Mean ± SD (SE) of duplicate determination. * Significantly different in relation to Control (* *p* ≤ 0.05; ** *p* ≤ 0.01). ^◊^ Significantly different in relation to Control + HT (^◊^ *p* ≤ 0.05; ^◊◊^ *p* ≤ 0.01). Abbreviations: Res—resveratrol solution at a dose of 50 mg/kg; Cis—cisplatin at doses of 2.5 and 5 mg/kg; Res + Cis—resveratrol solution at a dose of 50 mg/kg and cisplatin at doses of 2.5 and 5 mg/kg; HT—hyperthermia; SE—Standard error; SD—Standard deviation.

From Figure 6, it is evident that the concentration of VEGF is reduced in all groups compared to the Control. The smallest decrease of VEGF concentration was 11.5% (Control + HT), and the highest was 49.3% (Cis + HT). As expected, the combination of cisplatin with hyperthermia had a more pronounced inhibitory effect on VEGF concentration compared to cisplatin in physiological conditions (49.3% vs. 37.82%, respectively). A significant reduction of VEGF concentration compared to Control or Control + HT was demonstrated for groups Cis + HT (*p* < 0.01; *p* < 0.01), Res + Cis (*p* < 0.05; *p* < 0.05), and Res + Cis + HT (*p* < 0.05).

VEGF is the key factor in tumour neovascularization. It increases endothelial cell proliferation and migration, tumour growth in vivo, and remodels the perivascular matrix by increasing levels of proteinases, including matrix metalloproteinases MMP2 and MMP9 [2,3,4,5].

**Figure 7:**
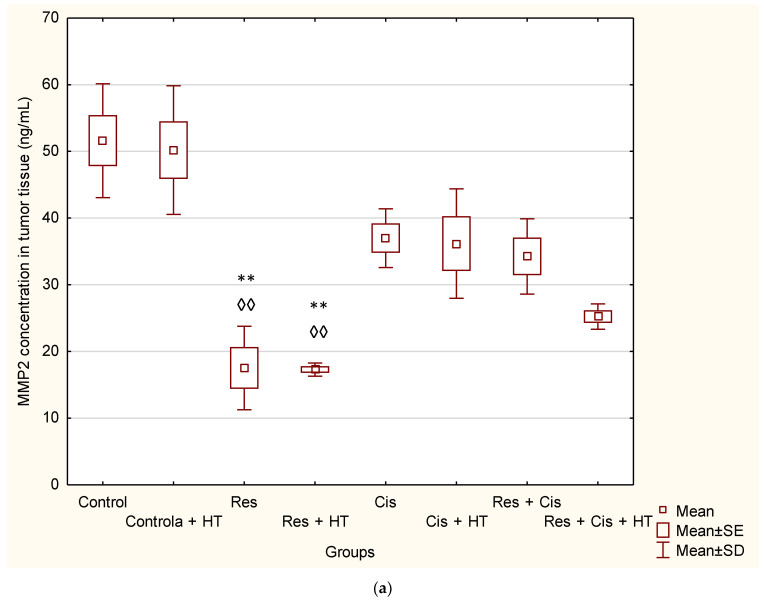

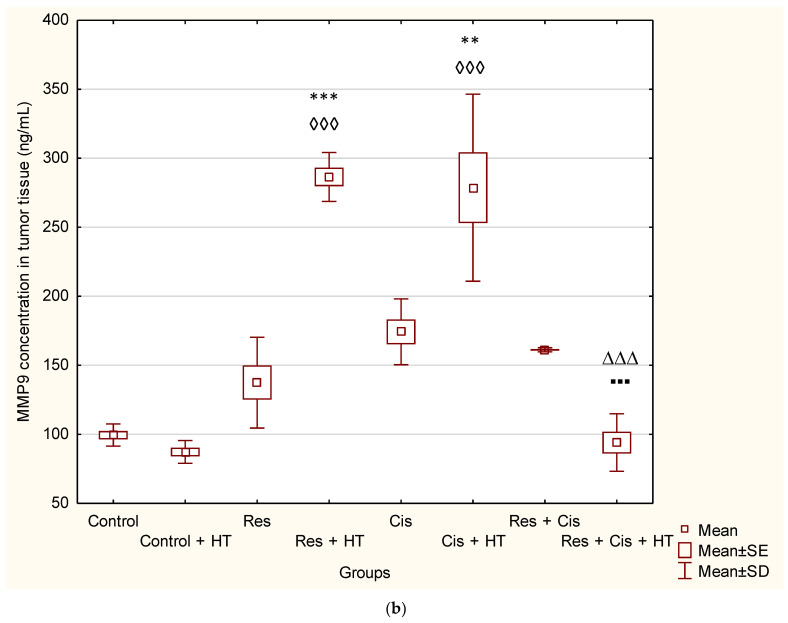
MMP2 (**a**) and MMP9 (**b**) concentrations in tumour tissue in mice bearing the solid form of EAT treated with resveratrol, cisplatin, and their combination in physiological and hyperthermic conditions. After subcutaneous (*sc*) injection of 1 × 10^6^ EAT cells, mice (N = 5 per group) were treated with a dose of 50 mg/kg resveratrol during five consecutive days starting from the second day after injection, while cisplatin was injected intraperitoneally (*ip*) at a dose of 2.5 mg/kg on days 10 and 12 and at a dose of 5 mg/kg on day 15. Groups treated with hyperthermia were exposed to systemic hyperthermia lasting for 15 min at a temperature of 41 °C. Obtained results are presented as Mean ± SD (SE) of duplicate determination. ^*^ Significantly different in relation to Control (** *p* ≤ 0.01; *** *p* ≤ 0.001). ^◊^ Significantly different in relation to Control + HT (^◊◊^ *p* ≤ 0.01; ^◊◊◊^ *p* ≤ 0.001). ^Δ^ Significantly different in relation to Res + HT (^ΔΔΔ^ *p* ≤ 0.001). ^■^ Significantly different in relation to Cis + HT (^■■■^ *p* ≤ 0.001). Abbreviations: Res—resveratrol solution at a dose of 50 mg/kg; Cis—cisplatin at doses of 2.5 and 5 mg/kg; Res + Cis—resveratrol solution at a dose of 50 mg/kg and cisplatin at doses of 2.5 and 5 mg/kg; HT—hyperthermia; SE—Standard error; SD—Standard deviation.

MMP2 concentration (Figure 7a) was reduced in all groups except in Control + HT. Interestingly, the highest effect was observed for Res with or without HT compared to Control (*p* < 0.01 in both groups).

Contrary to MMP2, the concentration of MMP9 (Figure 7b) was upregulated in almost all groups compared to the Control with/or without HT, especially in the Res + HT (*p* < 0.001 or *p* < 0.001) and Cis + HT groups (*p* < 0.001 or *p* < 0.01). MMP9 concentration in the group treated with Res + Cis + HT was significantly lower compared to the Res + HT and Cis + HT groups (*p* < 0.001 and *p* < 0.001). 

Tumour progression and the escape of tumour cells (metastasis) require a dynamic interaction between cancer cells and their microenvironment. The induction of tumour angiogenesis is a critical step in the early development of solid tumours. Formation of new blood vessels requires the angiogenic factor VEGF and its proteolytic release from tumour matrix by MMPs, predominantly by the MMP-9 that is delivered into the tumour microenvironment by tumour-infiltrating leukocytes. Given the reduced level of VEGF and tumour growth, we next investigated whether the number of blood vessels was also reduced within the solid form of EAT treated with resveratrol and cisplatin with or without hyperthermia. The obtained results clearly indicated that there was a significant inhibition of neovascularization in mice treated with Res (*p* < 0.05), Cis (*p* < 0.01), Cis + HT (*p* < 0.05), and Res + Cis + HT (*p* < 0.05) compared with the tumours of Control animals that showed extensive neovasculature (Table 3). However, the difference in microvessel density was not found between corresponding groups in physiological and hyperthermic conditions, including between Cis + HT and Res + Cis + HT groups.

Tumour progression is regulated by paracrine interactions between tumour and stromal cells. Nitric oxide (NO) plays an important role in the regulation of interactions between the tumour and its microenvironment, thereby affecting tumour progression [19]. The tumour microenvironment includes different types of cells such as fibroblasts, pericytes, immune cells, and tumour-associated macrophages (TAMs). According to numerous studies, NO can have a dual role. It may both promote and suppress tumour growth. We focused our research on the tumouricidal effect of macrophages, that is, the induction of M1 macrophages by analysing NO and arginase levels. High levels of NO are associated with the inhibition of tumour growth, while arginase levels are potential diagnostic biomarkers for cancer progression.

**Figure 8:**
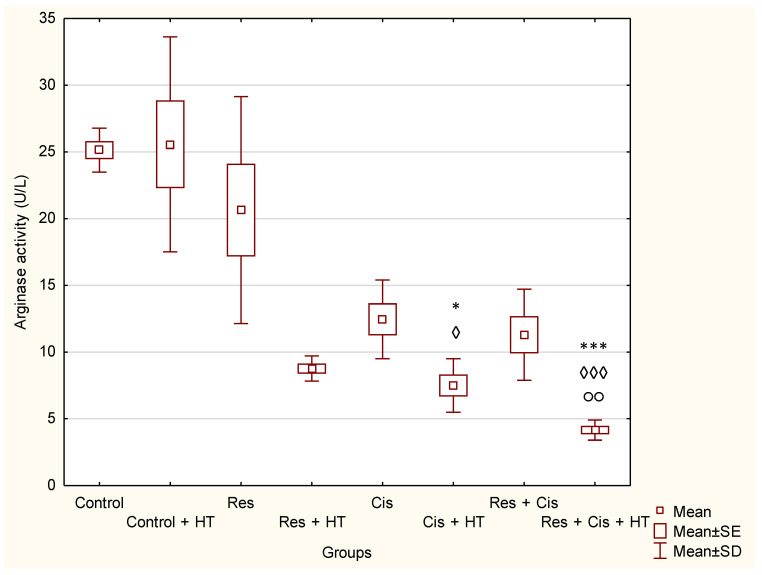
Arginase activity (U/L) in mice bearing the solid form of EAT treated with resveratrol, cisplatin, and their combination in physiological and hyperthermic conditions. After subcutaneous (*sc*) injection of 1 × 10^6^ EAT cells, mice (N = 5 per group) were treated with a dose of 50 mg/kg resveratrol during five consecutive days starting from the second day after injection, while cisplatin was injected intraperitoneally (*ip*) at a dose of 2.5 mg/kg on days 10 and 12 and at a dose of 5 mg/kg on day 15. Groups treated with hyperthermia were exposed to systemic hyperthermia lasting for 15 min at a temperature of 41 °C. Obtained results are presented as Mean ± SD (SE) of duplicate determination. * Significantly different in relation to Control (* *p* ≤ 0.05, *** *p* ≤ 0.001). ^◊^ Significantly different in relation to Control + HT (^◊^ *p* ≤ 0.05, ^◊◊◊^ *p* ≤ 0.001). ^○^ Significantly different in relation to Res (^○○^ *p* ≤ 0.01). Abbreviations: Res—resveratrol solution at a dose of 50 mg/kg; Cis—cisplatin at doses of 2.5 and 5 mg/kg; Res + Cis—resveratrol solution at a dose of 50 mg/kg and cisplatin at doses of 2.5 and 5 mg/kg; HT—hyperthermia; SE—Standard error; SD—Standard deviation.

Arg activity (Figure 8) in the tumour tissue was low in all groups treated with Cis (Cis, Cis + HT, Res + Cis, Res + Cis + HT), while statistical significance compared to the Control with or without HT was obtained in groups Cis + HT *(p* < 0.05; *p* < 0.05) and Re + Cis + HT (*p* < 0.001; *p* < 0.001).

**Figure 9:**
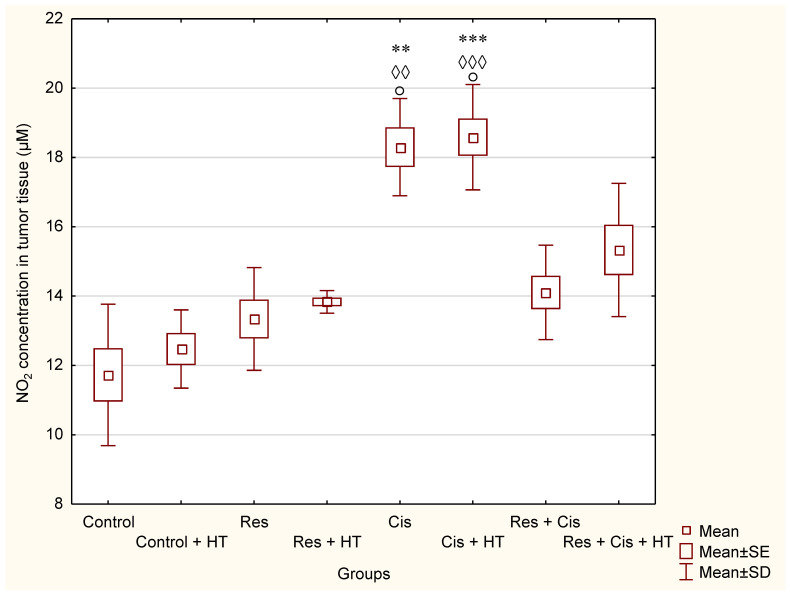
NO concentration in tumour tissue (µM) in mice bearing the solid form of EAT treated with resveratrol, cisplatin, and their combination in physiological and hyperthermic conditions. After subcutaneous (*sc*) injection of 1 × 10^6^ EAT cells, mice (N = 5 per group) were treated with a dose of 50 mg/kg resveratrol during five consecutive days starting from the second day after injection, while cisplatin was injected intraperitoneally (*ip*) at a dose of 2.5 mg/kg on days 10 and 12 and at a dose of 5 mg/kg on day 15. Groups treated with hyperthermia were exposed to systemic hyperthermia lasting for 15 min at a temperature of 41 °C. Obtained results are presented as Mean ± SD (SE) of duplicate determination. * Significantly different in relation to Control (** *p* ≤ 0.01; *** *p* ≤ 0.001). ^◊^ Significantly different in relation to Control + HT (^◊◊^ *p* ≤ 0.01; ^◊◊◊^ *p* ≤ 0.001). ^○^ Significantly different in relation to Res (^○^ *p* ≤ 0.01). Abbreviations: Res—resveratrol solution at a dose of 50 mg/kg; Cis—cisplatin at doses of 2.5 and 5 mg/kg; Res + Cis—resveratrol solution at a dose of 50 mg/kg and cisplatin at doses of 2.5 and 5 mg/kg; HT—hyperthermia; SE—Standard error; SD—Standard deviation.

For the same groups (Cis, Cis + HT, Res + Cis, Res + Cis + HT), the level of NO (Figure 9) was increased, especially in the Cis and Cis + HT group compared to the Control (*p* < 0.01; *p* < 0.01), Control + HT (*p* < 0.001; *p* < 0.001), and Res (*p* < 0.05).

### 2.4. Combination of Resveratrol, Cisplatin, and Hyperthermia Induces Histological and Immunohistochemical Changes in Tumour Tissue of Balb/c Mice Bearing the Solid Form of EAT

**Figure 10:**
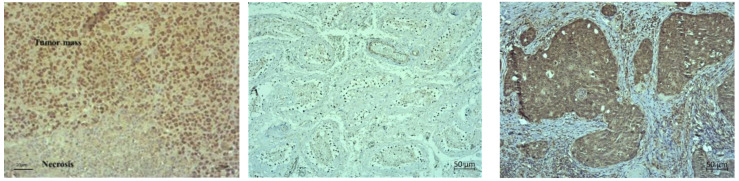
Immunohistochemical staining of the paraffin-embedded solid form of EAT using proliferating cell nuclear antigen (PCNA) mouse antibody (Dako, M0879) at a dilution 1:100 (magnification 200×). Brown staining is visible in the nuclei of tumour cells. Highly proliferating tumour cells are located peripherally in the tumour mass, while the centre of the tumour is necrotic. Considering that all samples showed the same profile of very high proliferation (over 90%), we showed only one representative photomicrograph. Immunohistochemistry of the paraffin-embedded testis tissue stained using HIF-1α, Rabbit pAb (ABclonal, A16873) at a dilution 1:100 (magnification 100×) is presented in the middle, and immunohistochemistry of the paraffin-embedded breast cancer stained using iNOS, rabbit pAB at a dilution 1:120 (Abcam, ab3523) is presented on the right (magnification 100×). Representative photomicrographs of immunohistochemical slides were taken.

**Figure 11:**
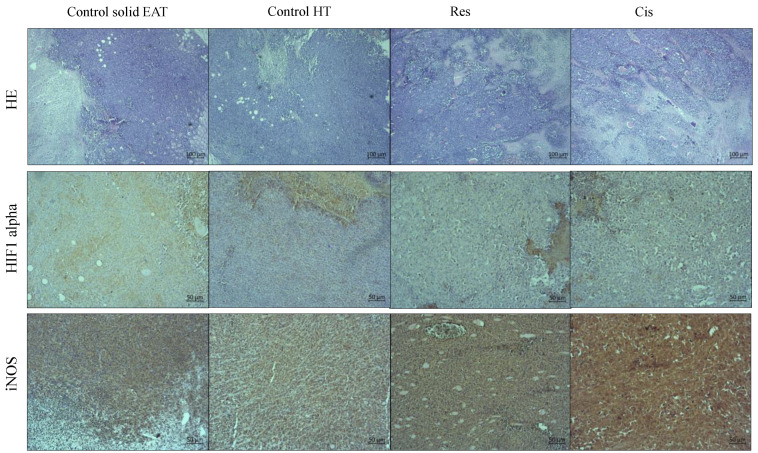
Photomicrographs of the histology of control samples treated with one agent (N = 5) as well as immunohistochemical reactions for hypoxia inducible factor 1 alpha (HIF-1α) and inducible nitric oxide synthase (iNOS). HIF-1α expression showed weaker cytoplasmic expression without any visible nuclear staining. The immunohistochemical reaction of iNOS showed a moderate cytoplasmic reaction. Representative photomicrographs of immunohistochemical slides were taken. Abbreviations: Res—resveratrol solution at a dose of 50 mg/kg; Cis—cisplatin at doses of 2.5 and 5 mg/kg; HT—hyperthermia; HE—hematoxylin and eosin.

**Figure 12:**
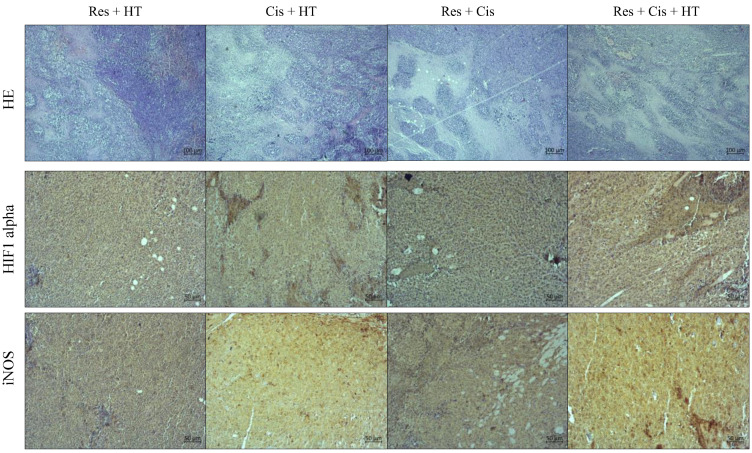
Photomicrographs of the histology of samples treated with combined agents (N = 5) as well as immunohistochemical reactions for hypoxia inducible factor 1 alpha (HIF-1α) and inducible nitric oxide synthase (iNOS). HIF-1α expression showed moderate cytoplasmic expression in Res + Cis and Cis + HT groups with sporadic nuclear staining in Res + Cis + HT treated group. The immunohistochemical reaction of iNOS showed moderate cytoplasmic reaction more expressed in Cis + HT and Res + HT treated groups. Representative photomicrographs of immunohistochemical slides were taken. Abbreviations: Res—resveratrol solution at a dose of 50 mg/kg; Cis—cisplatin at doses of 2.5 and 5 mg/kg; Res + Cis—resveratrol solution at a dose of 50 mg/kg and cisplatin at doses of 2.5 and 5 mg/kg; HT—hyperthermia; HE—hematoxylin and eosin.

The pathological examination of the paraffin-embedded solid EAT samples showed visible central necrosis in all specimens, the degree of which cannot be assessed (Figure 10, first rows on Figure 11 and Figure 12). In all samples, the tumour cells were located at the periphery of the tumour mass and showed a very high degree of proliferation (more than 90%). Therefore, in Figure 10 we showed one representative example of nuclear positivity for the proliferating cell nuclear antigen (PCNA). However, it is clearly visible that the treated samples showed different patterns of necrosis (Figure 11 and Figure 12). For the single-agent treated samples, we observed the most extensive necrosis following treatment with Cis and then Res.

Hypoxia inducible factors 1 and 2 (HIF) are important transcriptional regulators of angiogenesis and energy metabolism in tumours and an important target for cancer therapy [20]. Accumulating evidence indicates that inhibitors of histone deacetylases repress the function of HIF in tumour cells through yet unclear pathways. Based on the above, we investigated HIF activity, as well as the key factors of angiogenesis (VEGF, MMP2, and MMP9) and the number of blood vessels.

After immunohistochemical staining, we observed weak cytoplasmic staining for HIF-1α in control and single-agent treated samples (Figure 11). Although the solid EAT tumour (Control) showed focal cytoplasmic staining, we did not observe any nuclear staining indicative of transcriptional activation of hypoxia-activated signalling pathways. Testicular cancer was used as a positive control for hypoxia-inducible factor 1 (HIF-1α), with clear nuclear positivity (Figure 10).

Immunohistochemical staining for inducible nitric oxide synthase (iNOS) showed weak to moderate cytoplasmic staining for iNOS in control and single-agent treated samples. The strongest expression was observed in samples treated with Cis. Breast cancer was used as a positive control for iNOS with strong cytoplasmic staining (Figure 10). 

After immunohistochemical staining, we observed moderate cytoplasmic staining for HIF-1α in all samples treated with combined agents (Figure 12). The staining was more intense than the Control or single-agent treated samples. However, moderate cytoplasmic staining with sporadic stained nuclei was observed in Res + Cis + HT and Cis + HT groups while some sporadic stained nuclei were observed only in Res + Cis + HT, compared to staining in all other treatment groups.

The immunohistochemical staining for iNOS also showed moderate cytoplasmic staining in all samples treated with combined agents. The strongest expression of iNOS was observed in samples treated with Cis + HT and Res + Cis + HT.

**Figure 13:**
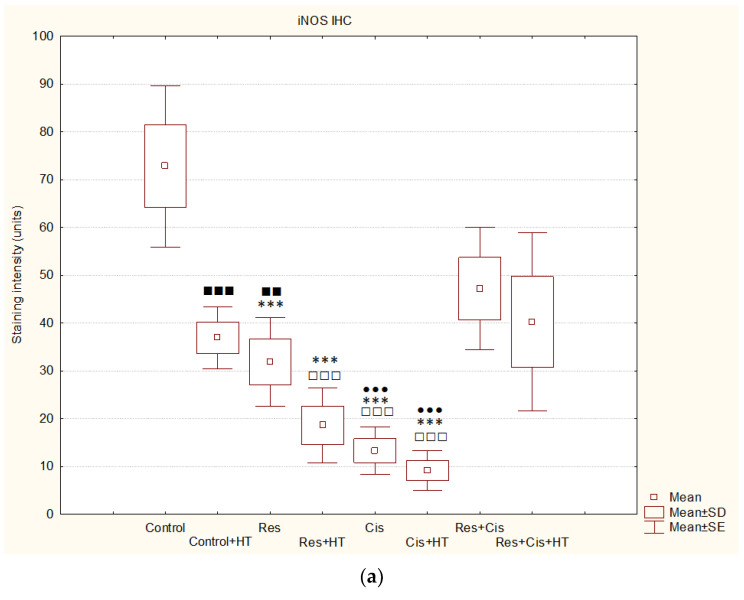

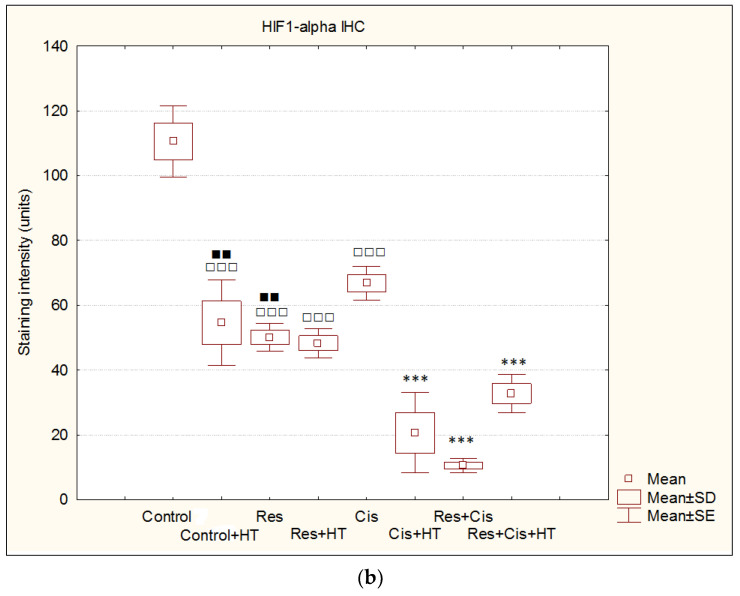
Immunohistochemical staining intensity of iNOS (**a**) and HIF-1α (**b**) presented as units (BN value) transformed using the mathematical equation published by Fu et al. [21] to solve the overlapping RGB colour spectrum of the stains. Most DAB-stained pixels have low BN values, which are lower as the brown colour becomes darker. Hematoxylin-stained negative control had the median intensity value of 203 units (not presented). (**a**) iNOS: Tumour tissue (Control) without any experimental treatment showed the weakest iNOS IHC expression with 72.3 ± 8.6 units. The strongest staining intensity of iNOS showed tumour tissue of animals treated with Cis + HT (9.17 ± 2.1), Cis (13.3 ± 2.5), and Res + HT (18.6 ± 4.0). (**b**) HIF-1α: Tumour tissue (Control) without any experimental treatment showed the weakest HIF1-alpha IHC expression with 110.5 ± 5.6 units. The strongest staining intensity of iNOS showed tumour tissue of animals treated with Res + Cis (10.6 ± 1.0), Cis + HT (20.7 ± 6.3), and Res + Cis + HT (32.8 ± 3.0). For IHC analysis, tumour tissue samples were taken from five animals per group and at least five immunostained preparations per one tumour tissue sample were analysed (N = 25 samples per group).* Significantly different in relation to Control (*** *p* ≤ 0.001). ^■^ Significantly different in relation to Cis + HT (^■■^ *p* ≤ 0.01, ^■■■^ *p* ≤ 0.001). ^□^ Significantly different in relation to Res + Cis (^□□□^ *p* ≤ 0.001). ^●^ Significantly different in relation to Res + Cis + HT (^●●●^ *p* ≤ 0.001). Abbreviations: Res—resveratrol solution at a dose of 50 mg/kg; Cis—cisplatin at doses of 2.5 and 5 mg/kg; Res + Cis—resveratrol solution at a dose of 50 mg/kg and cisplatin at doses of 2.5 and 5 mg/kg; HT—hyperthermia; SE—Standard error; SD—Standard deviation.

For the quantitative analysis of the intensity of immunohistochemical staining of the images represented in Figure 11 and Figure 12, we used the computer-assisted method described by Fu et al. [21]. The obtained data presented as units (BN value) showed that the strongest iNOS staining intensity was visible in the tumour tissue of animals treated with Cis + HT (9.17 ± 2.1), Cis (13.3 ± 2.5) and Res + HT (18.6 ± 4.0) (Figure 13a). Treatment of animals with Cis and Cis + HT showed the greatest changes of iNOS levels. The statistical difference was obtained in comparison to Control (*p* ≤ 0.001), Res + Cis (*p* ≤ 0.001), and Res + Cis + HT goups (*p* ≤ 0.001), while the treatment of animals with Res + HT was statistically different compared to Control and Res + Cis (*p* ≤ 0.001 for both groups). In addition, changes in the level of iNOS were noticed in the Res group compared to Control (*p* ≤ 0.001) and Cis + HT (*p* ≤ 0.01), and between Control + HT vs. Cis + HT (*p* ≤ 0.01). Quantitative analysis of HIF-1α showed statistically significant changes in the group treated with Cis + HT, Res + Cis, and Res + Cis + HT compared to the Control (in all group *p* ≤ 0.001). Immunohistochemical staining intensity presented as units (BN value) of HIF-1 in tumour tissue of these groups was as follows: 10.6 ± 1.0 (Res + Cis), 20.7 ± 6.3 (Cis + HT), and 32.8 ± 3.0 (Res + Cis + HT), while the staining intensity in the tumour Control tissue was 110.5 ± 5.6 units (Figure 13b). A statistical difference was also found between the Res + Cis group and the Control +HT, Res, and Res + HT and Cis groups (*p* ≤ 0.001), as well as between Cis + HT vs. Control + HT and Res (*p* ≤ 0.01). 

## 3. Discussion

Tumour hyperthermia is a powerful therapeutic approach and chemotherapy sensitizer. It increases the cytotoxicity of cancer cells and stimulates the immune response through the activation of immune cells. Hyperthermia modulates local pro-inflammatory responses, immune activation, cell death, and microenvironmental changes at target sites, mainly by specific heat shock proteins (HSPs) [6,7,8,9,18]. In addition, several studies have demonstrated that hyperthermia may be useful in overcoming resistance to chemotherapeutic drugs such as cisplatin by increasing drug uptake and by changing tumour microcirculation, blood flow, cell membrane permeability, and cellular metabolism [6,7,8,9,10,11,12]. Almost all methods of tumour therapy can cause some side effects, it is considered that the application of natural antioxidants, present ©n food and dietary supplements, with a pronounced chemopreventive activity can reduce the effects of toxicity on healthy cells and increase the antitumour effectiveness of chemotherapeutic drugs such as cisplatin.

Previous studies have shown that resveratrol can increase the antitumour efficacy of cisplatin and reverse multidrug resistance by increasing the chemosensitivity of cancer cells [9,11,12]. Additionally, resveratrol as an HDAC inhibitor can induce cancer cell cycle arrest, differentiation, and death, reduce angiogenesis, and modulate the immune response, thus opening up new perspectives for its clinical use as a chemopreventive drug or cancer therapeutic [11,12]. 

In this study, we investigated whether the pre-treatment with the natural antioxidant resveratrol, in combination with systemic hyperthermia and cisplatin, can enhance the effect of cisplatin on tumour cells through the modulation of HDAC activity and the HSP70 and HSP90 levels and consequently contribute to direct toxicity on tumour cells, the inhibition of angiogenesis and tumour growth, and ultimately increase the lifespan of mice.

Our research clearly indicates that hyperthermia increases cisplatin toxicity, reduces body weight, and improves treatment outcomes by inhibiting angiogenesis and tumour growth, leading to the increased life span of mice (Figure 1, Figure 2 and Figure 3, Table 1). However, the loss of tumour mass was not exclusively related to the toxicity of cisplatin, but also to the inhibition of tumour growth and angiogenesis. The results showed decreased concentration of VEGF in tumour cells in all treated groups, especially in the group treated with Cis + HT, where the value was statistically significant (*p* < 0.01) compared to the Control (Figure 6). This group (Cis + HT) had the smallest tumour volume compared to other groups (Figure 3) and the highest percentage of tumour growth inhibition (Table 1), together with the significantly suppressed HDAC activity (*p* < 0.001) in relation to Control (Figure 4). Similar results were shown for the group treated with Res + Cis + HT regarding the inhibition of tumour growth, %ILS, VEGF level, and HDAC activity (Table 1, Figure 1, Figure 2, Figure 3, Figure 4 and Figure 6). It seems that the multimodal approach of the combination of resveratrol and cisplatin with hyperthermia increases antitumour efficacy, which is in accordance with our previous data and other literature data [6,7,8,9].

The formation and maintenance of blood vessel structures are essential for the physiological functions of tissues and important for the progression of diseases such as cancer. Without adequate vascular supply, solid tumours can grow only to a critical size of 1–2 mm, primarily due to the lack of oxygen and nutrients. The growth of solid tumours depends on the formation of new blood vessels (angiogenesis), affecting the size of the tumour and the process of metastasis, which are closely related to the levels of VEGF, MMP-2, and MMP-9 [8,9].

Hyperthermia with Cis can inhibit VEGF production in vivo and shows the anti-angiogenic effect, which was confirmed by the reduced number of blood vessels (MVD) (Table 3). Hence, the exposure of malignant cells to chemotherapeutic drugs and hyperthermia can be a therapeutic strategy for the prevention of tumour progression and inhibition of angiogenesis by interfering with cell replication and/or inhibition of the migration of vascular endothelial cells. It is likely that the microvessel damage may play an important role in the tumouricidal effect of hyperthermia and Cis as most of the tumour cells died after microvessel damage in vivo. It has been confirmed that in the initial phase of hyperthermia cell death is mainly induced by apoptosis, while later the rate of necrosis increases [9], as shown by our histological preparations (Figure 9, Figure 10 and Figure 11). Dilation, congestion, and rupture of tumour microvessels after hyperthermia may reduce tumour blood flow and cause an inadequate supply of nutrients and oxygen and acid accumulation, whereas low pH leads to tumour growth control, which is evident in the significantly prolonged survival and inhibition of tumour growth.

By binding to the VEGF receptor on endothelial cells, VEGF stimulates the secretion of MMPs and induces disruption of the extracellular matrix [9]. Substrates for MMPs can be various precursors and receptors of growth factors, adhesion receptors, and cytokines and their receptors [22]. Increased levels of MMP-2 and MMP-9 are associated with the invasive and metastatic potential of malignant ovarian tumours [1,2,3,4,5,8,9,22]. Likewise, it has been shown that resveratrol may reduce the expression level of MMP-2 and MMP-9 proteins in MDA-MB-231 cells [23]. The reduced levels of MMP-2 and MMP-9 result in reduced invasiveness and metastasis, and consequently, a better prognosis [1,2,3,4,5]. Our results showed the significant decrease in the concentration of MMP-2 in the group treated with Res and Res + HT compared to the Control (*p* < 0.01; *p* < 0.01). In other treated groups, the concentration of MMP-2 was also lower compared to the Control, but without significance (Figure 7a). The decrease in the concentration of MMP-2 in tumour cells after treatment with resveratrol is in accordance with the results of the research conducted by Gagliano et al. [24], which demonstrated the ability of resveratrol to reduce MMP-2 expression in primary glioblastoma cell culture. Additionally, Xiong et al. [25] showed that resveratrol can inhibit the motility and invasiveness of glioblastoma cells by reducing the expression of MMP-2.

Furthermore, resveratrol inhibited the migration and invasion of prostate cancer cells by promoting demethylation and increasing the expression of tissue inhibitors of metalloproteinases, TIMP2, and TIMP3 and by suppressing the expression of MMP-2 and MMP-9 [26,27]. Contrary to these findings, our results show a significantly higher concentration of MMP-9 in the group treated with Res + HT and Cis + HT compared to the Control group (*p* < 0.001; *p* < 0.01) (Figure 7b). Additionally, the Res + HT group has an increased concentration compared to the Res + Cis + HT group (*p* < 0.001) (Figure 7b). The obtained results can be explained by the dual role of MMPs. Specifically, MMPs are involved in numerous processes, including growth, survival, angiogenesis, invasion, and immunity, but also in the control of cell death, inflammation, infection, and anti-angiogenesis. Although lower levels of MMP-9 are associated with a better prognosis of tumour treatment, higher levels do not necessarily mean the opposite effect, given the role of MMP-9 in the control of angiogenesis and its ability to cleave anti-angiogenic precursors and activate angiostatin and other anti-angiogenic factors such as endostatin and tumstatin, which consequently reduces tumour growth and blood vessel density [28]. Angiostatin, as an anti-angiogenic factor, reduces the proliferation and migration of endothelial cells. It is released from plasminogen by the action of plasmin, plasmin reductase, and several MMPs (MMP-12, MMP-9, MMP-7, and MMP-3) [1,2,3,4,5]. Whereas VEGF levels within the tumour environment appear to directly correlate with the overall microvessel density, specific inhibition or trapping of VEGF produced by cancer cells results in a significant reduction of tumour angiogenesis, concomitant with reduced tumour growth and, consequently, the inhibition of metastasis. Our results show that resveratrol and cisplatin have an anti-angiogenic effect (Table 3), which is partly related to the inhibition of VEGF and/or MMP-2 activity and possibly through the production of angiostatin via MMP-9 activity.

Activation of VEGF as a pro-angiogenic factor is mediated by HIF-1α to induce angiogenesis. Hypoxic conditions also regulate HDAC activity directly or indirectly through the HDAC involvement in oxygen-regulated gene expression and hypoxia-induced angiogenesis [10,19]. An important finding of our research is that the combination of Cis, HT, and resveratrol significantly suppresses HDAC activity, blocks the binding of HIF-1α to DNA, and modulates levels of HSP70 and HSP90 (Figure 4 and Figure 5). Our data indicate that the suppression of HIF-1α activity and the impairment of tumour cell-induced angiogenesis is mediated through the inhibition of HIF-1α translocation into the nucleus. These data are consistent with the findings of Liang et al. [20], who suggested that HDCIs have anti-HIF activity through the destabilization of HIF-1α and the repression of the transactivation potential of the C-terminal activating domain of HIF-α (HIF-αCAD). Other known mechanisms of anti-HIF activity are the repression of DNA binding ability and the inhibition of nuclear translocation of HIF-1α. Quantitative analysis of HIF-1α showed higher levels in Res + Cis, Cis + HT, and Res + Cis +HT groups, indicating increased cell death by apoptosis or necrosis, which is in good agreement with the reduced tumour volume and increased animal survival (Figure 13b). 

Cells respond to stressful conditions by activating stress response proteins, which may be important in maintaining a fine balance between cell death and survival. Stress proteins induced after chemotherapy, radiation, or hyperthermia can have a dual role. Thus, HSPs may display cytoprotective functions due to the ability to protect cells from damaging stress, acting as survival regulators through their anti-apoptotic properties [20]. On the other hand, HSPs may be involved in antigen processing and presentation and can act as “danger signals” for the adaptive and innate immune systems. More specifically, in immunosuppressive conditions, the HSPs enhance the survival and proliferation of cancer cells by activating their cellular protection machinery, whereas under optimal conditions HSPs may stimulate the anticancer immune response [6,7,20].

The reduced level of HSP90 in all groups, except for the control groups in physiological and hyperthermic conditions and resveratrol, confirms the sensitivity of tumour cells to the inhibition of HSP90 (Figure 5b), whose expression is generally increased in tumours compared to normal cells. It is known that one of the side effects of HSP90 inhibition is the compensatory induction of HSP70, which is also indicated by our results (Figure 5a).

The Induction of protective antitumour immunity and its manifestations at local and distal sites result from a complex interplay between different cell types and soluble mediators such as cytokines and stress proteins. An increasing number of studies have shown that hyperthermia, as an adjuvant approach to cancer immunotherapy, correlates with the increased presence of infiltrating monocytes and macrophages in tumours, and that this tends to be associated with increased macrophage activity [6,29,30,31,32,33] and tumour regression [5,29,30,31,32,33]. An increase in the immunomodulatory activity of macrophages after hyperthermia and resveratrol was also evident in our study (Figure 8 and Figure 9), indicating the importance of the immune system in tumour rejection. The possible molecular mechanisms underlying the improved immune reactivity in the presence of hyperthermia include the generation of HSPs, the activation of antigen presenting cells, and changes in lymphocyte trafficking. As mentioned, HSPs are involved in antigen processing and presentation and can act as “danger signals”. The immunogenic nature of HSP-70 relies on its ability to bind antigenic tumour peptides [24,29,30,31,32,33]. The HSP70-peptide complex results in an antigen-specific CD8+ T cell response. To recognize tumour cell antigens, CD8+ T cells require the expression of MHC class I antigens, the expression of which is low or completely abolished in as many as 40–90% of primary tumours and metastases. This mechanism helps tumours to avoid an immune reaction to the tumour. The lack of the MHC-class I molecules makes tumour cells “invisible” to the cytotoxic action of CD8+ T cells. On the contrary, the lack of MHC expression makes tumours more sensitive to a certain subset of natural killer (NK) cells. Activated NK cells, which express HSP70 receptors such as the C-type lectin receptors CD94/NKG2C and NKG2D, bind to membrane HSP70 on tumour cells and secrete granzyme B, either alone or in combination with perforin, leading to cell death [20].

It is known that chemotherapeutic “drugs” such as cisplatin, doxorubicin, mitomycin C, 5-fluorouracil, and camptothecin, which exert direct cytotoxic effects, also exhibit an indirect cytotoxic activity by “preparing” tumour cells for elimination by immune cells such as NK or cytotoxic T lymphocytes using a Fas or TNF-related apoptosis-inducing ligand (TRAIL)-dependent pathway [34]. Chemotherapy can also enhance the immune response against cancer by modifying the interaction between tumour cells and dendritic cells (DC), which play a key role in antigen presentation to T lymphocytes. In addition, chemotherapy can enhance the endocytosis of tumour cells by dendritic cells. Finally, tumour cells are involved in the production of “danger signals” that are required for dendritic cell maturation and tumour antigen presentation to T cells [6,29,30,31,32,33]. It has been theorized that following tumour heating, absorbed fragments of necrotic cancer cells provide the antigenic stimulus needed to enhance the host immune system, which then promotes the destruction of tumour cells [29,30,31,32,33,34]. In this way, hyperthermia may have either a direct suppressive effect on tumour cells by inhibiting tumour cell proliferation, and by inducing cell arrest, apoptosis, and microvessel damage, or it may act indirectly by enhancing host immune function [6,7,8,34]. Cancer immunotherapy has tremendous potential, but it has yet to be clinically proven in various tumour situations. Hence, strategies capable of inducing immunogenic cell death (e.g., cisplatin, hyperthermia) together with the reprogramming of the immunosuppressive tumour microenvironment (e.g., M2-to-M1-like macrophages repolarization of tumour-associated macrophages (TAMs)), are particularly appealing for enhancing the efficacy of approved immunotherapies (e.g., immune checkpoint inhibitors). TAMs are an important part of solid tumours. In some tumours, almost 40% of non-malignant cells are TAMs [35]. Namely, several chemotherapy- and hyperthermia-mediated mechanisms can be involved in the antitumour response, from the activation of immune cells via HSPs and “dangers signals” to the elimination of immunosuppressive cells. Some of the most important mechanisms include the disturbance of immunosuppression mediated by Treg cells and myeloid suppressor cells, strengthening the maturation of dendritic cells and their functions through the presentation of tumour antigens, promoting the activity of cytotoxic T cells, NK cells, and macrophages, increasing the penetration of immune cells into the tumour core, remodelling the tumour microenvironment, and enhancing antigen display by tumour cells that increases their susceptibility to the programmed immune clearance. 

Thus, platinum-based chemotherapies, including cisplatin, exert immunogenic effects through the cell death induction and the release of death-associated molecular patterns, which activate pro-inflammatory signalling pathways. It has been reported that cisplatin-induced macrophage activation stimulates the synthesis and release of NO, tumour necrosis factor (TNF)-α, interleukin (IL)-1, interferon (IFN)-γ, and IL-12, leading to M1 polarisation [32,33,34]. In addition, the maturation of DCs in the presence of platinum drugs downregulates their levels of PD-L1 and PD-L2 and increases the potential for T-cell activation [32]. Likewise, it was shown that resveratrol plays a similar role in the induction of immunogenic cell death through the expression of calreticulin (CRT) and high mobility group box 1 (HMGB1) on the surface of ovarian cancer cells and results in the significant inhibition of tumour volume and suppression of the tumour growth factor (TGF)-β while promoting levels of IL-12 and IFN-γ [11,17,18,26]. Resveratrol enhances antitumour T cell immunity by promoting abnormal glycosylation and dimerization of PD-L1, preventing PD-1 interaction with the PD-L1, and, consequently, increasing the susceptibility of aggressive cancer cells to T-cell-mediated cell death. Evidently, synergism in multimodal therapy is crucial for the induction of immunogenic cell death through the activation of macrophages and other immune cells (Figure 8 and Figure 9). 

The activation of M1 macrophages induces the production of NO. An increased concentration of NO has a cytotoxic effect on tumour cells and promotes apoptosis. M1 macrophages increase the activity of the iNOS enzyme, which catalyses the conversion of L-arginine to NO and citrulline, while M2 macrophages increase the expression of the Arg-1 enzyme. Hence, these two enzymes are in competition for L-arginine [36,37]. M1 macrophages are pro-inflammatory. They inhibit tumour cell proliferation and destroy damaged tissues by secreting pro-inflammatory cytokines (IL-12 and IL-23) and reactive oxygen species (ROS) and NO, and they have a high ability to present antigens [36,37]. It has been shown that NO production by the tumour microenvironment is essential for the antitumour activity of CD8+ T-cells [38]. NO upregulates death receptors and enhances their antitumour and cytotoxic activity [39]. The effects of NO in cancer gained attention after the observation that activated macrophages metabolize arginine to generate NO, an effector molecule that induces cytotoxicity in tumour cells [37,38,39,40]. NO produced by NOS2-expressing myeloid cells acts together with CD8+ T-cells to eliminate tumour cells. Therefore, identifying NO’s role in tumour immune response is essential for developing efficient immunotherapies that target diverse aspects of NO metabolism. The role of NO in tumours depends on NO concentration, the duration of exposure, cell sensitivity, extracellular conditions, and iNOS localization in tissues [41,42,43]. Low and medium concentrations of NO promote angiogenesis, metastases, and invasiveness, and inhibit apoptotic processes. At higher concentrations, NO becomes cytotoxic to tumour cells and promotes apoptosis [44]. Our data are consistent with the aforementioned results [37,38,39,40,41,42,43,44]. We confirmed the reactivation of TAM macrophages towards the M1 phenotype in all groups treated with Cis (Cis, Cis + HT, Res + Cis, Res + Cis + HT), while Arg activity was found to be reduced (Figure 8 and Figure 9). The increased level of iNOS was confirmed in all treated groups, especially in the Res, Cis + HT, Cis, and Res + HT groups (Figure 13a). Overexpression of iNOS in TAMs was associated with a favorable prognosis in breast and lung cancer, a high degree of apoptosis, and decreased post-therapy outbreak of the disease [12,37,38,39,40,41,42,43,44].

On the other hand, M2 macrophages have an anti-inflammatory and immunosuppressive effect, with a low ability to present antigens. They promote tumour growth and angiogenesis [45]. An increasing number of studies have shown that arginase 1 and 2 play a key role in the regulation of tumour growth and metastasis through various mechanisms, including the regulation of L-arginine metabolism, the modulation of signalling pathways, and influencing the tumour microenvironment. Therefore, arginase is appreciated as an attractive target of cancer therapy. Arginase regulates the immune escape of cancer cells through several mechanisms: (i) high expression of Arg-1 and Arg-2 in cancer cells directly impairs the function of T cells by depleting L-arginine in the tumour microenvironment; (ii) secreted Arg-1 or extracellular vesicles containing Arg-1 from myeloid-derived suppressor cells (MDSCs) and TAMs deplete L-arginine levels in tumour microenvironment and suppress T-cell activation and proliferation; (iii) finally, the high expression of Arg-2 in Treg cells enhances the suppressive capacity and accumulation of Treg cells via the inhibition of the mTOR signalling pathway, which in turn enables the immune escape of tumour cells [46,47,48,49,50,51]. Our results showed significantly reduced activity of Arg-1 in groups treated with Cis + HT and Res + Cis + HT (*p* < 0.001) compared to the Control group (Figure 8). The reduced activity of Arg-1 in these groups indicates the polarization of M2 macrophages towards the M1 phenotype, which results in better antitumour effects and increased life span (Figure 2, Figure 3, Figure 8 and Figure 9, Table 1 and Table 2).

According to our data, cisplatin, as one of the most important and most frequently used chemotherapeutic agents, can increase the effector activity of cytotoxic T cells and NK cells by inhibiting Treg and MDSC cells. A synergism between the resveratrol, cisplatin, and hyperthermia promotes the death of immunogenic tumour cells through the induction of damage-associated molecular patterns (DAMPs) such as CRT, HSP70, and HMGB1, and via the remodelling of the tumour microenvironment by the increasing maturation of DCs and macrophages. Activated DCs and macrophages, as antigen presenting cells, reactivate the effector function of cytotoxic T cells on tumour cells, as well as the activation of NK cells by HSP70 [20,29,30,31,32,33,34], leading to tumour growth inhibition and lifespan increase (Figure 1, Figure 2 and Figure 3 and Figure 5).

It should also be noted that resveratrol is one of the most important dietary constituents capable of chemoprevention and vasculoprotection.

Aging induces numerous changes such as genomic instability, epigenetic alterations, telomere shortening, loss of proteostasis, deregulation of nutrient sensing, mitochondrial dysfunction, cellular senescence, and stem cell exhaustion. Various changes induced by microenvironmental factors and aging-dependent changes are partially related to blood vessels, which are important for the maintenance of tissue function and the transport of oxygen, food, and waste materials. During aging, the density of blood vessels and the number of pericytes decrease [52,53], together with the decrease of NO production that increases apoptosis of endothelial cells [54]. Furthermore, pericytes are a source of fibroblasts in inflammatory processes and the differentiation of pericytes into fibroblasts increases with aging and aging-related diseases, including tumours. Cancer-associated fibroblasts (CAFs), together with other tumour-associated cells, support tumour growth and metastasis by inducing immunosuppression. Besides that, normal human aging is characterized by a progressive decline in immune surveillance that is accompanied by an increase in PD-L1, which consequently leads to tumour initiation and progression. Resveratrol inhibits the ROS—NF-κB axis, supresses platelet activation, increases NO bioavailability, and/or activates sirtuins, thus helping in the preservation of a youthful vascular phenotype and the prevention of cellular senescence [55]. Furthermore, resveratrol, as a naturally occurring PD-1/PD-L1 immune checkpoint inhibitor, may directly block the enzymatic machinery involved in N-linked glycosylation of the nascent PD-L1 at the endoplasmic reticulum or directly bind to PD-L1 surfaces to induce PD-L1 dimerization and block PD-1 binding. The suppression of PD-L1 signalling may be an immunometabolic mechanism through which resveratrol can prevent immune dysfunction and cancer development in the context of aging [56].

It seems that HIF inhibition with resveratrol ultimately prevents resistance to anti-angiogenic therapy, blocks epithelial-mesenchymal transition and differentiation of myeloid suppressive cells into M2-proangiogenic TAM, contributes to the reactivation of NK and T cell activity, and prevents genetic instability in tumour endothelial cells and the selection of invasive metastatic tumour cell clones that are resistant to anti-angiogenic agents [11,53,57,58]. The indirect effect of resveratrol on angiogenesis is based on the increase of p53 activity in a dose-dependent manner in both cancerous and non-cancerous cell lines through the up-regulation of anti-angiogenic factors, including thrombospondin-1 and the reduction of VEGF expression in cancer cells. Thus, a multi-modal approach and the ability to target several cancer-related mechanisms with multiple drugs would be an ideal approach for controlling tumour cell proliferation/stemness, angiogenesis, chronic inflammation, and immune tolerance.

In conclusion, resveratrol can enhance the antitumour effect of cisplatin by modulating HDAC activity and HSP70 and HSP90 levels, thus contributing to direct toxicity on tumour cells and tumour growth, and consequently prolonging mice life. Furthermore, it seems that hyperthermia can create a favourable tumour microenvironment for an immune chain reaction that improves the efficacy of the combined treatment with cisplatin and resveratrol. Hyperthermia enhances local tumour blood flow and increases the accumulation of cisplatin within tumour tissues and remodulates the tumour microenvironment by realizing danger signals and antigens originating from tumour cells that contribute to the reactivation of macrophage polarization into the M1 phenotype and the activation of the immune response to tumour cells. Summarizing the data, treatments of animals with Cis and HT and Res + Cis + HT have the greatest effects in antitumour activity, but apparently via the slightly different synergistic/antagonistic mechanisms. According to the obtained results, the use of resveratrol did not significantly contribute to the antitumour effect of cisplatin and hyperthermia, but it partially contributed to minor differences in animal survival, MMP2 concentration, and a significant decrease in arginase activity, which is important in HIF-NO signalling. With an increased understanding of the mechanisms underlying these processes, more accurate and effective therapies can be developed for the growing number of diseases influenced by HIF-NO signalling. It is possible that the microenvironmental conditions of the tumour, especially the availability of oxygen, affects the redox balance of Res and changes its chemotoxic activity, but also weakens the activity of cisplatin. Although the use of natural components in combination with cisplatin often gives an advantage in attenuating tumour growth, angiogenesis, chronic inflammation, and immune tolerance, thus reducing the consequences of toxicity, caution is needed when using multimodal therapy because the microenvironmental conditions of the tumour can significantly affect the final outcome of tumour therapy. However, the precise mechanism of the interaction between resveratrol, cisplatin, and HT remains unclear and needs to be investigated further. 

## 4. Material and Methods

### 4.1. Experimental Animals and Ethics

The research was conducted on inbred Balb/c mice obtained from the Department of Animal Physiology, Faculty of Science, University of Zagreb. The mice were 2–3 months old, weighing 20–25 g. A total of 120 animals were divided into 8 groups of 15 animals. They were fed standard food for laboratory animals (4RF 21 Mucedola S.R.L., Settimo Milanese MI, Italy; Batch No. 238603, shape 12 mm) with constant access to water. The conditions were standard and included a daily rhythm of 12 h of light and 12 h of darkness and a temperature of 24 °C with controlled air humidity. The study was approved by the Ethics Committee (Faculty of Science, University of Zagreb, Croatia; approval code: 251-58-10617-19-1150 of 5 December 2019) and was conducted in accordance with the ethical principles valid in the Republic Croatia (Law on Animal Welfare, NN 102/2017) [59]; Law on Amendments to the Law on Animal Welfare, NN 37/13 [60]; Regulation on the Protection of Animals Used for Scientific Purposes, NN 55/13 [59]) and according to the Guide for the Care and Use of Laboratory Animals, DHHS (NIH) Publ # 86-23, National Research Council [61].

### 4.2. Tumour Cells

Ehrlich’s ascites tumour (EAT) is an undifferentiated carcinoma originating from mouse mammary cells. It is hyperdiploid, highly transmissible, fast-growing, completely malignant with extremely sensitive and heterogeneous cells, and lacks tumour-specific transplantation antigens (TSTA), which makes it a valuable model for research. EAT is very similar to human tumours that are most susceptible to chemotherapy since it is an undifferentiated, angiogenesis-dependent, fast-growing tumour and as such is suitable for studying the effect of chemical and natural compounds on tumour growth, angiogenesis, inflammation, and immunomodulation.

EAT cells were maintained intraperitoneally (*ip*) in Balb/c mice by serial transplantation of cells every 7 or 9 days in the form of ascites. After washing the abdominal cavity with 5 mL of saline and gently massaging the abdominal wall, an extensive incision was performed to open the peritoneal cavity of the mouse. Using a Pasteur pipette, peritoneal fluid with tumour cells was taken and diluted with physiological solution (0.9% sodium chloride solution, Pliva, Zagreb, Croatia) to the final concentration of EAT cells (1 × 10^6^ cells in 0.5 mL/mouse). The number of living cells was determined by staining with trypan blue in a Bürker–Türk chamber. In this study, we used a solid model of tumour growth caused by the subcutaneous (*sc*) application of EAT cells into the right femoral region of the mouse.

### 4.3. Resveratrol

The resveratrol solution was prepared by dissolving resveratrol (Evolva SA, Reinach, Switzerland) in water with the addition of DMSO. The solution was administered per os (*po*) by intragastric cannula at a dose of 50 mg/kg.

### 4.4. Cisplatin

The cytostatic agent cisplatin (Cisplatin, Pliva, Zagreb, Croatia) was prepared immediately before use in a dose of 5 mg/kg by diluting it in water for injections (Aqua pro injectione, Pliva, Zagreb, Croatia) and injected in a dose of 2.5 mg/kg *ip* for 10 days and 12 days and in dose of 5 mg/kg for 15 days.

### 4.5. Experimental Design, Animal Treatment, and Organ Processing

Mice were injected with 1 × 10^6^ EAT cells subcutaneously in the right femur to induce Ehrlich solid tumour formation. Before and during the experiment, the weight of the animals was individually measured, and the dose of test components was adjusted according to the mice’s daily weight. All animals were divided into two large groups (Group I—physiological conditions, Group II—hyperthermal conditions), each containing 4 groups, (N = 15 animals in each group). After the injection of tumour cells, the animals were intragastrically (*ig*) treated with resveratrol at a dose of 50 mg/kg for 5 consecutive days, starting 2 days after the tumour injection. The treatment with resveratrol started after the injection of the tumour, considering that resveratrol is a common flavonoid present in many plants (more than 70 plant species) and wine. Cisplatin was injected three times [16]: on day 10 after the injection of tumour cells when the tumour became visible (approximately 1000 mm^3^) and on day 12 in a dose of 2.5 mg/kg, while the third dose of 5 mg/kg was injected on day 15 with or without hyperthermia (Table 4). Twenty-four hours later the animals were sacrificed. Group II was exposed to systemic hyperthermia according to the protocol of Duhan et al. [62], with small changes in temperature and exposure time. The animals were exposed to heating at 41 °C for 15 min.

**Figure 14:**
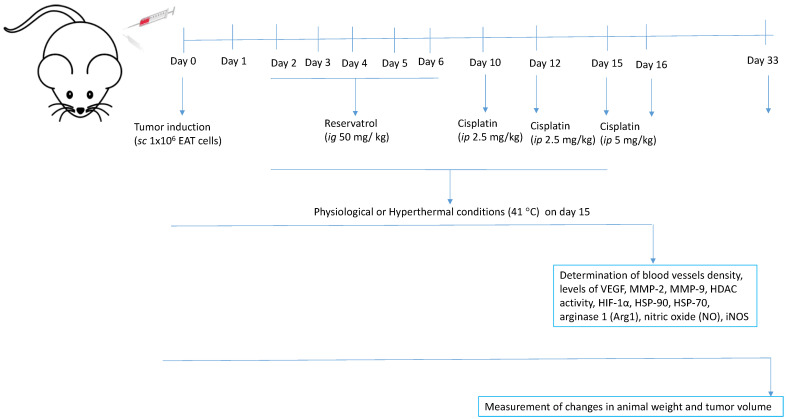
Experimental design, animal treatment, and tumour tissue analysis.

After treatment, 5 animals from each group were sacrificed and tumour tissue was taken for the analysis of angiogenesis parameters (number of blood vessels, levels of VEGF, MMP-2, MMP-9, HDAC activity, HIF-1α, HSP-90, HSP-70, nitric oxide (NO) arginase 1 (Arg1). During tumour collection, all animals were adequately anesthetized by intraperitoneal administration of a combination of Narketana^®^ Vetoquinol S.A., BP 189 Lure Cedex, France (active substance Ketamin) and Xylapana^®^ Vetoquinol Biowet Sp., Gorzow, R. Poland (active substance Xylazine) in a dose of 25 mg/kg. The rest of the animals (*n* = 10) were used to monitor the growth and size of the tumour with a caliper and to determine the weight of the animals and the increase in life span and overall survival up to three months (see Figure 14).

### 4.6. Monitoring the Dynamic of Tumour Growth and the Survival of Experimental Animals

When monitoring tumour growth and the survival of the animals, the instructions of the “Guide for the Care and Use of Laboratory Animals” and all the rules related to the experimental and human endpoints of the experiment were respected. The animals were monitored daily by an appointed veterinarian or professionally trained PhD student. With the indication of the first disturbances in appearance, behaviour, and pain, the animals were sacrificed; the longest period of keeping the animals was three months.

#### 4.6.1. Percentage Increase in Life Span

In the survival studies, each group contained 10 randomly selected mice and the survival of mice was recorded. Increased life span (% ILS) was calculated according to the following formula:

% ILS = [(T − C)/C] × 100%;

T—mean value of the survival time of the treated animals; C—the mean survival time of the control animals. 

#### 4.6.2. Measurement of Changes in Animal Weight and Tumour Volume

Tumour growth and the effects of therapy at the level of the whole organism were determined by weighing the animals during the experiment and monitoring the mass change. Animals were weighed on a digital balance (Kern KB 2000-2N P.b.: 0.01 g: 2000 g) every 7 days. The weight loss or gain of the animals compared to the Control group was monitored. Tumour volume was measured with a caliper according to the formula:

Tumour volume (mm^3^) = [(tumour width)^2^ × tumour length)]/2

From the obtained data, % tumour growth inhibition (%TVI) was calculated according to the formula:

%TV I = [(Mean tumour vol._Control_ − Mean tumour vol._Treted group_) × 100)/(Mean tumour vol._Control_)]

### 4.7. HDAC Activity in Tumour Tissue

HDAC activity was assessed using a colorimetric HDAC assay (HDAC Activity Colorimetric Assay Kit, Biovision, Waltham, MA, USA). Before HDAC analysis, nuclear extraction of tumour tissue was performed, and protein concentration was determined according to Bradford.

The method was performed according to the manufacturer’s instructions. The analysis is based on two steps. First, a colorimetric HDAC substrate (containing an acetylated lysine side chain) was incubated with a sample that has HDAC activity. By deacetylation, the substrate becomes more sensitive, so in the second step, treatment with a lysine developer produces a chromophore, which was analysed with a microtiter reader at 450 nm.

For the standard curve, the deacetylated standard from the assay was diluted and concentrations of 100 μM, 50 μM, and 25 μM were used, whereas for 0 μg/μL, 90 μL of dH_2_O and 10 μL of 10× HDAC buffer were prepared.

Briefly, 10 μL of the sample and 75 μL of dH_2_O were added to the corresponding wells. The positive control contained 10 μL of HeLa nuclear extract in 75 μL ddH_2_O, and the sample itself in 83 μL ddH_2_O with the addition of 2 μL trichostatin was used as the negative control. Then 10 μL of 10× HDAC buffer and 5 μL of colorimetric HDAC substrate were added to the wells. The plate with the prepared samples was incubated for 1 h at 37 °C. After incubation, 10 μL of lysine developer was added and incubated again at 37 °C for 30 min. Absorbance was measured using a reader at λ = 400 nm, and concentrations were determined and calculated using a standard curve.

### 4.8. Determination of HSP-70 and HSP-90 Levels in Tumour Tissue

Tumour HSP-70 and HSP-90 levels were determined using a commercial mouse ELISA kit (MyBioSource, San Diego, CA, USA) according to the manufacturer’s instructions. HSP-70 and -90 are expressed as pg/mg tissue protein. Briefly, 50 μL of the standard or sample (tumour homogenates) or 50 μL of PBS was added to the wells as a negative control. Then, 5 μL of the “balance” solution was added to the sample wells with mixing, and 100 μL of the conjugate was added to each well (except for the negative control). The samples were then incubated for 1 h at 37 °C. After washing, 50 μL of substrate A and 50 μL of substrate B were added to the wells and additionally incubated for 20 min at 37 °C. Stop solution (50 μL) was added to stop the reaction. Absorbance (λ = 450 nm) was measured using a microtiter plate reader, and the concentration of HSP-70 was calculated using the standard curve.

For HSP-90, the standard and samples were added to the corresponding wells at 100 μL. The microtiter plate with samples was incubated for 90 min at 37 °C. After washing the wells with washing buffer (2×), 100 μL of biotin-labelled antibody was added and incubated for 60 min at 37 °C. After incubation and washing the wells again (3×), 100 μL of SABC solution (containing HRP-streptavidin) was added and the plate was incubated for 30 min at 37 °C. The wells were then washed 5 times, and 90 μL of 3,3′,5,5′-Tetramethylbenzidine (TMB) substrate was added and incubated in the dark at 37 °C for about 15 min. At the end of the incubation period, the reaction was stopped by adding 50 μL stop solution and the absorbance was measured at λ = 450 nm using a microreader (Ao Absorbance Microplate Reader, Azure Biosystems, Dublin, CA, USA).

### 4.9. Determination of the Levels of MMP-2 and MMP-9

The levels of MMP-2 and MMP-9 in the tumour cell supernatant were determined using the Mouse MMP-2 ELISA Kit and the Mouse MMP-9 ELISA Kit manufactured by Chongqing Biospes Co., Ltd., Biospes, (Chongqing, China) according to the manufacturer’s instructions. The method is based on the sandwich ELISA technique. Anti-MMP-2 or anti-MMP-9 polyclonal antibodies were previously applied to a 96-well plate. Biotin conjugated anti-MMP-2 and anti-MMP-9 polyclonal antibodies were used to detect bound metalloproteinases. Exactly 100 µL of the standard, sample, and control were added to the wells of the microtiter plate in the indicated order and incubated at 37 °C for 90 min, whereby MMP-2 or MMP-9 binds to the immobilized antibody. After removing the contents of all wells, 100 µL of Biotin-conjugated anti-mouse MMP-2 or MMP-9 antibody was added to all wells and the samples were incubated at 37 °C for 60 min. After washing the wells with washing buffer, 100 µL of ABC (avidin-biotin-peroxidase) complex was added and the samples were incubated at 37 °C for 30 min. This was followed by repeated washing (5×) of samples with washing buffer and the addition of 100 µL of TMB substrate to visualize the HRP enzymatic reaction and the formation of a blue colour by incubating at 37 °C in the dark for 30 min. The reaction was stopped by adding 0.1 mL of stop solution to each well, causing the colour to turn to yellow. The yellow intensity is proportional to the amount of mouse MMP-2 or MMP-9 bound at the beginning of the reaction. Absorbance values were read at 450 nm on a Microplate reader Model 550, Bio-Rad company. The standard for MMP-2 was made according to the manufacturer’s instructions in the concentration range from 0 pg/mL to 20,000 pg/mL, while the standard for the determination of MMP-9 was in the concentration range from 0 pg/mL to 5000 pg/mL. Concentrations of MMP-2 or MMP-9 were calculated using a standard curve. The samples were run in triplicate. The sensitivity of this ELISA kit for determining the concentration of mouse MMP-2 is <10 pg/mL, while for MMP-9 it is <20 pg/mL.

### 4.10. Determination of VEGF Level

The mouse VEGF Quantikine^®^ ELISA kit (R&D Systems, Inc., Minneapolis, MN, USA) was used to determine the concentration of the VEGF. All solutions, standards, controls, and samples were prepared according to the instructions in the kit. Briefly, 50 μL of RD1N solution was added to each well, and then a standard, control, or corresponding sample was applied to the corresponding wells in a volume of 50 μL. Incubation lasted for 2 h at room temperature. After washing, 100 μL of mouse VEGF conjugate was added to the wells and incubated again for 2 h at room temperature. After washing, substrate solution (100 μL) was added and incubated for 30 min in the dark at room temperature. “Stop” solution (100 μL) was added to stop the reaction. Absorbance values were read at 450 nm with the wavelength correction at 540 nm. The VEGF standard (500 pg/mL) from the kit was dissolved with “Calibrator Diluent RD5T” buffer in the concentration range from 0 to 250 pg/mL. From the standard curve of dependence of absorbance on the concentration of the standard VEGF solution, the concentration of VEGF in the tested samples was determined and expressed in pg/mL.

### 4.11. Estimation of Nitric Oxide (NO)

The Griess reagent system kit manufactured by Promega (Madison, WI, USA), which uses a 1% sulfanilamide solution prepared in 5% phosphoric acid and a 0.1% solution of N-1-naphthylethylenediamine dihydrochloride (NED) prepared in water, was applied for nitrite analysis. Nitric (II) oxide concentration was measured on homogenized tumour samples. Sodium nitrite dissolved in water at concentrations from 0 to 100 μM was used as a standard. From the standard curve of the dependence of the absorbance on the nitrite concentration, the slope of the line was determined, and the concentration of NO_2_^−^ in the samples, expressed as μM/μL, was calculated from the slope.

### 4.12. Arginase Activity Assay

Arginase activity assay kit MAK112 (Sigma-Aldrich, Saint Louis, MO, USA) was used to measure the level of arginase 1 activity from a tumour tissue and was performed according to the manufacturer’s instructions. Arginase is an enzyme that catalyses the conversion of L-arginine to L-ornithine and urea, whereby the produced urea reacts specifically with the substrate to produce a colour proportional to the activity of arginase. The absorbance was measured at 430 nm (Ao Absorbance Microplate Reader, Azure Biosystems, Dublin, CA, USA). One unit of arginase represents the amount of enzyme sufficient to convert 1 μM L-arginine to ornithine and urea per minute at pH 9.5 and 37 °C. Arginase activity was determined using the following formula:

Arginase activity = [A(sample) − A(empty well)]/[A(standard) − A(water)] × [(1 mM × 50 × 10^3^)/(V × T)] where T is the reaction time in minutes, V is the volume of the sample added to the wells, 1 mM is the concentration of urea standard, 50 is the reaction volume, and 10^3^ is the conversion factor from mM to μM.

### 4.13. Histological Analysis of the Tumour

After animal sacrifice, Erlich’s solid tumour mass was collected from the mouse thigh and placed in 10% buffered formalin for 48 h, after which the tissue was embedded in paraffin by routine laboratory procedure (FFPE procedure). Tissue sections 3–5 μm thick were cut from paraffin blocks, mounted on glass slides, and stained with hematoxylin and eosin for pathological examination.

#### 4.13.1. Immunohistochemical Staining of Tumour Tissue

Several sections were cut and placed on positively charged slides for immunohistochemical staining. Antigen unmasking was conducted by heat treatment in the PTLink module using Dako 3 in 1 Antigen retrieval buffer pH 9.0 (EnVision^TM^FLEX Target Retrieval Solution, High pH, K8000). The following primary antibodies were used for immunohistochemical staining: (a) Hypoxia-inducible factor 1 alpha (HIF1α, Rabbit pAB, dilution 1:100; aBclonal, cat no. A16873); (b) Inducible Nitric Oxyde Synthase (iNOS, rabbit pAB, dilution 1:120; Abcam, cat no. ab3523); (c) Proliferating Cell Nuclear Antibody (PCNA, mouse mAB, 1:100; Dako, cat no. M0879). Primary antibodies against HIF1α and PCNA were applied on tumour tissue for 2 h at room temperature, while iNOS was applied overnight at 4 °C. After washing with wash buffer, the secondary antibody, which is part of DAKO’s EnVision^TM^ FLEX/HRP detection kit (DAKO K8000), was applied for 45 min at room temperature. This kit used to visualize the reaction contains peroxidase, secondary antibodies, chromogen, and wash buffer. Immunohistochemical staining was performed in automated Dako Autostainer Link 48. The sections were contrast-stained with hematoxylin. Representative photomicrographs of immunohistochemical slides were taken.

Quantitative information of the immunohistochemical reaction was expressed as the percentage of the positively stained area, the intensity of the brown stain in the positive areas, and morphological characteristics. Since the investigated proteins HIF1-alpha and iNOS showed ubiquitous distribution in all cells, we used digital image analysis to quantify the IHC staining intensity [21]. 

At least ten digital photographs of each IHC tissue sample were taken with a light microscope at magnification of 200×, and the digital analysis was performed using Zeiss ZEN Lite, software version 2.3. In microscopic images, the colour of each pixel consists of three 8-bit monochrome channels (Red, Green, and Blue; RGB) resulting in a 24-bit colour image. Areas of cancerous tissues that show a positive immunohistochemical reaction have brown pigmentation. The median intensity of the RGB signal was read on a completely taken image using the Histogram option in the program, and the median intensity was recorded for each individual colour of the RGB spectrum. The brown colour consists mostly of the Red but also a part of the Green as well as Blue contrasting colouring, so we used the mathematical equation published by Fu et al. [21] to solve the problem of the overlapping colour spectrum of the stains. They used this mathematical model to convert RGB images to 8-bit BN images using the formula:

BN = Blue − α × Red − β × Green

According to their publication, when the α to β ratio is two, the images produce better contrast between brown DAB-stained tissues and blue hematoxylin-stained tissues. Therefore, we also used α = 0.5 and β = 0.25. Most DAB-stained pixels have low BN values, which are lower as the brown colour becomes darker.

#### 4.13.2. Determination of Blood Vessel Density in Tumour Tissue

Small blood vessel density (MVD) was determined according to Weidner’s method [63]. The number of blood vessels was counted in about 20 hotspot fields throughout the tumour area, at a magnification of 200× on a Zeiss Axio Star microscope with a field of view of 1 mm.

### 4.14. Statistical Analysis

For an estimation of the minimum sample size required for the experiment, STATA 13.1 (StataCorp. 2013. Stata Statistical Software: Release 13. College Station, TX, USA: StataCorp LP.) was used. Using a non-parametric Kruskall–Wallis rank sum test with an alpha level of 0.05, a power level of 0.8, and an effect size of 0.22, the estimated minimum sample size was 15 mice per treatment group. Statistical analyses were performed using STATISTICA 13 software (StatSoft, Tulsa, OK, USA) and data were presented as mean ± standard error (SE) of the mean. All data were analysed through a Kruskal–Wallis ANOVA test and further analyses of the differences between the groups were made with multiple comparisons of mean ranks for all groups. 

The data were considered significant at *p* < 0.05. Treatment-dose specific survival curves were calculated by the Kaplan–Meier method, and comparison between the survival curves was made by log-rank test (α = 5%) as described in [6,7,17]. 

## Figures and Tables

**Table 1:** Survival range, mean survival time, and increased life span (%) in mice bearing the solid form of EAT treated with resveratrol, cisplatin, and their combination in physiological and hyperthermic conditions.

Experimental Group ^a^	Survival Range (Days)	Mean Survival (Days)	Median (Days)	% T/C ^b^	% ILS ^c^	Number of Surviving Animals	Tumour Growth Inhibition (% TVI) vs. Physiological Control		Tumour Growth Inhibition (% TVI) vs. Hyperthermic Control	
							on Day 16	on Day 33	on Day 16	on Day 33
Control	22–38	30.44 ± 1.79	32.00	/	/	0	/	/	/	/
Control + HT	28–40	34.77 ± 1.10	34.00	114.22	14.22	0	21.78	18.10	/	/
Res	30–55	39.90 ± 2.14	39.50	131.07	31.07	0	17.43	34.06	−5.56	19.48
Res + HT	35–45	40.00 ± 0.97	41.00	131.40	31.40	0	33.62	24.79	15.13	8.17
Cis	21–70	38.90 ± 4.60	36.00	127.79	27.79	0	52.77	41.95	39.62	29.12
Cis + HT	34–80	51.11 ± 6.28	45.00	167.90	67.90	1	64.02	51.59	54.01	40.89
Res + Cis	30–49	41.33 ± 2.59	46.00	135.77	35.77	0	51.26	52.87	37.69	42.45
Res + Cis + HT	32–96	51.30 ± 6.37	45.00	168.52	68.52	1	56.47	41.75	44.35	28.88

^a^ After subcutaneous (*sc*) injection of 1 × 10^6^ EAT cells, mice (N = 10 per group) were treated with a dose of 50 mg/kg resveratrol during five consecutive days starting from the second day after injection, while cisplatin was injected intraperitoneally (*ip*) at a dose of 2.5 mg/kg on days 10 and 12 and at a dose of 5 mg/kg on day 15. Groups treated with hyperthermia were exposed to systemic hyperthermia lasting for 15 min at a temperature of 41 °C. Obtained results are presented as Mean ± SE. ^b^ T/C% = T/C × 100; T—mean survival days of treated groups; C—mean survival days of Control group. ^c^ %ILS (increased life span %) = (T − C)/C × 100. Abbreviations: Res—resveratrol solution at a dose of 50 mg/kg; Cis—cisplatin at doses of 2.5 and 5 mg/kg; Res + Cis—resveratrol solution at a dose of 50 mg/kg and cisplatin at doses of 2.5 and 5 mg/kg; HT—hyperthermia; SE—Standard error.

**Table 2:** Survival comparison in mice bearing the solid form of EAT treated with resveratrol, cisplatin, and their combination in physiological and hyperthermic conditions.

Experimental Group ^a^	% ILS Physiological Conditions	% ILS Hyperthermic Conditions	Kaplan–Meier Analysis (Log-Rank Test)	
			* *p*	^◊^ *p*
Control	-		-	0.1093
Control + HT	14.22	-	0.1093	-
Res	31.07	14.75	0.00933	0.03685
Res + HT	31.40	15.04	0.00309	0.00659
Cis	27.79	11.88	0.08076	0.2149
Cis + HT	67.90	46.99	0.00541	0.01821
Res + Cis	35.77	18.87	0.01241	0.06584
Res + Cis + HT	68.52	47.54	0.00233	0.00506

^a^ After subcutaneous (*sc*) injection of 1 × 10^6^ EAT cells, mice (N = 10 per group) were treated with a dose of 50 mg/kg resveratrol during five consecutive days starting from the second day after injection, while cisplatin was injected intraperitoneally (*ip*) at a dose of 2.5 mg/kg on days 10 and 12 and at a dose of 5 mg/kg on day 15. Groups treated with hyperthermia were exposed to systemic hyperthermia lasting for 15 min at a temperature of 41 °C. Survival rates were calculated using the Kaplan–Meier method and comparison between the survival curves was made by log-rank test (α = 5%). * Significantly different in relation to Control (log-rank test). ^◊^ Significantly different in relation to Control + HT (log-rank test). Abbreviations: Res—resveratrol solution at a dose of 50 mg/kg; Cis—cisplatin at doses of 2.5 and 5 mg/kg; Res + Cis—resveratrol solution at dose of 50 mg/kg and cisplatin at doses of 2.5 and 5 mg/kg; HT—hyperthermia; %ILS (increased life span %) = (T − C)/C × 100.

**Table 3:** Microvessel density count (MVD) in mice bearing the solid form of EAT treated with resveratrol, cisplatin, and their combination in physiological and hyperthermic conditions.

Experimental Groups ^a^	Number of Blood Vessels (Mean ± SE)	Range	
		Minimum	Maximum
Control	11.14 ± 0.90	6	21
Control + HT	9.36 ± 0.74	4	18
Res	7.21 ± 0.71 *	3	14
Res + HT	7.76 ± 0.77	3	15
Cis	6.82 ± 0.49 **	2	13
Cis + HT	6.89 ± 0.51 *	2	10
Res + Cis	8.21 ± 0.78	2	14
Res + Cis + HT	6.78 ± 0.57 *	2	11

^a^ After subcutaneous (*sc*) injection of 1 × 10^6^ EAT cells, mice (N = 5 per group) were treated with a dose of 50 mg/kg resveratrol during five consecutive days starting from the second day after injection, while cisplatin was injected intraperitoneally (*ip*) at a dose of 2.5 mg/kg on days 10 and 12 and at a dose of 5 mg/kg on day 15. Groups treated with hyperthermia were exposed to systemic hyperthermia lasting for 15 min at a temperature of 41 °C. Individual microvessels were counted in the three areas of the highest vascular density on a 200× field (20 objective and 10 ocular). The microvessel density was expressed as the mean number of vessels in these areas [Mean ± SE (range)]. * Significantly different in relation to Control (* *p* ≤ 0.05; ** *p* ≤ 0.01). Abbreviations: Res—resveratrol solution at a dose of 50 mg/kg; Cis—cisplatin at doses of 2.5 and 5 mg/kg; Res + Cis—resveratrol solution at a dose of 50 mg/kg and cisplatin at doses of 2.5 and 5 mg/kg; HT—hyperthermia; SE—Standard error.

**Table 4:** Experimental design.

Group I—Physiological Conditions	Group II—Hyperthermal Conditions (HT)
1. Control	1. Control + HT
2. Resveratrol (50 mg/kg *ig*)	2. Resveratrol (50 mg/kg *ig*) + HT
3. Cisplatin (2.5 and 5 mg/kg *ip*)	3. Cisplatin (2.5 and 5 mg/kg *ip*) + HT
4. Resveratrol + Cisplatin	4. Resveratrol + Cisplatin + HT

## Data Availability

The original contributions generated for this study are included in the article; further inquiries can be directed to the corresponding author.

## Supplementary Materials

The following supporting information can be downloaded at: https://www.mdpi.com/article/10.3390/ijms241311073/s1.
