# Supplementary material for: The Role of Hyperthermia in Potentiation of Anti-Angiogenic Effect of Cisplatin and Resveratrol in Mice Bearing Solid Form of Ehrlich Ascites Tumour
Source: Int J Mol Sci. 2023 Jul 4;24(13):11073. doi: 10.3390/ijms241311073 (PMC10341868; doi:10.3390/ijms241311073)
Supplement: Supplementary file 1 [file ijms-24-11073-s001.zip › ijms-2405711-supplementary.pdf]

Table S1. Kaplan-Meier analysis of mice bearing solid form of EAT treated with resveratrol, cisplatin and their combination in physiological and hyperthermic conditions between all experimental groups.

Experimental group[a]	Kaplan-Meier analysis (log-rank test)							
	Control	Control + HT	Res	Res + HT	Cis	Cis + HT	Res + Cis	Res + Cis + HT
Control	-							
Control + HT	0.1093	-						
Res	0.00933	0.03685	-					
Res + HT	0.00309	0.00659	0.8969	-				
Cis	0.08076	0.2149	0.8710	0.70502	-			
Cis + HT	0.00541	0.01821	0.09024	0.13948	0.14906	-		
Res + Cis	0.01241	0.06584	0.50451	0.12846	0.95195	0,32635	-	
Res + Cis + HT	0.00233	0.00506	0.04231	0.02158	0.1830	0.68082	0.6316	-

[a]After subcutaneous (*sc*) injection of 1x10^6 EAT cells, mice (N=10 per group) were treated with the dose of 50 mg/kg resveratrol during 5 consecutive days starting from the second day after injection, while cisplatin was injected intraperitoneally (ip) at a dose of 2.5 mg/kg on days 10 and 12, and at a dose of 5 mg/kg on day 15. Groups treated with hyperthermia were exposed to system hyperthermia lasting for 15 minutes at the temperature of 41 °C. Survival rates were calculated using the Kaplan-Meier method and comparison between the survival curves was made by log-rank test (α = 5%).

Abbreviations: Res - resveratrol solution at a dose of 50 mg/kg; Cis - cisplatin at a dose of 2.5 and 5 mg/kg; Res + Cis - resveratrol solution at a dose of 50 mg/kg and cisplatin at a dose of 2.5 and 5 mg/kg; HT - hyperthermia; %ILS (increased life span %) = (T–C)/C×100 .
